# Producing mixed linked xylooligosaccharides from red algae biomass through single-step enzymatic hydrolysis

**DOI:** 10.1186/s13068-025-02686-7

**Published:** 2025-08-05

**Authors:** Michelle Teune, Christoph Suster, Yannick Wolf, Nils Michels, Henrieke Mieth, Thorben Döhler, Daniel Bartosik, Joris Krull, Jan-Hendrik Hehemann, Thomas Schweder, Christian Stanetty, Uwe T. Bornscheuer

**Affiliations:** 1 Department of Biotechnology & Enzyme Catalysis, Institute of Biochemistry, University of Greifswald, 17489 Greifswald, Germany; 2 Institute of Applied Synthetic Chemistry, TU Wien, 1060 Vienna, Austria; 3 Department of Pharmaceutical Biotechnology, Institute of Pharmacy, University of Greifswald, 17489 Greifswald, Germany; 4 Max Planck Institute for Marine Microbiology, 28359 Bremen, Germany; 5 Department of Biology/Chemistry, University of Bremen, 28359 Bremen, Germany

**Keywords:** Biomass, Glycoside hydrolase, Mixed-linkage, *Palmaria palmata*, Red algae, Xylan, Xylanase, Xylooligosaccharides

## Abstract

**Background:**

The red alga *Palmaria palmata* is a rich source of sugar compounds, particularly mixed-linkage xylans present in the cell walls of the algae. In contrast to their terrestrial lignocellulosic counterparts, these xylans are more easily accessible. They can be hydrolyzed enzymatically into valuable xylooligosaccharides (XOS), known for their prebiotic, antioxidant, and immunomodulatory properties.

**Results:**

This study introduces a simplified, one-step enzymatic process utilizing the *endo*-1,4-*β*-xylanase FO15_GH10 that directly hydrolyzes *P. palmata* biomass to produce XOS, eliminating the need for prior xylan extraction and improving efficiency. The exact structure of the resulting XOS was determined using NMR and MS/MS techniques. In addition, the xylosidase FO17_GH43 can be added to break down all residual 1,4-linked XOS. As a result, only 1,3- and mixed-linkage XOS (degree of polymerization (DP) 2–4) remains under simultaneous increase of the xylose obtained. Using FO15_GH10 alone, it was possible to produce approximately 17.6 (± 0.16) % (176 mg) XOS from 1 g of powdered biomass while combining both enzymes resulted in 22.6 (± 0.51) % (226 mg) XOS. Further optimization upon upscaling offers the possibility of achieving even greater improvements.

**Conclusion:**

In summary, our one-step enzymatic approach offers an efficient and sustainable method for producing XOS directly from *P. palmata* biomass. This streamlined process overcomes the need for resource-consuming extraction processes. The further characterization of the obtained XOS and the potential to gain solely 1,3- and mixed-linkage XOS is paving the way for future studies on their functional properties.

**Supplementary Information:**

The online version contains supplementary material available at 10.1186/s13068-025-02686-7.

## Background

The utilization of biomass, from terrestrial or marine origin, as a source of valuable compounds has been a major focus in biotechnology, particularly for applications in fuels, chemicals, foods, pharmaceuticals, or energy production [1–6]. Among other algae species, the red algae *Palmaria palmata* (dulse) has emerged as a promising source for biomass utilization due to its rich composition of bioactive molecules such as carotenoids, (iso-)floridosides, fatty acids, peptides, phenolic acids, and polysaccharides [7]. Especially the xylans, located in the cell walls of the macroalgae, are of interest. Their *β*-1,3-/1,4-mixed-linkage (1:4 ratio of *β*-1,3- and *β*-1,4) and loose hydrogen bonding in the cell walls are making them easier to extract than their terrestrial lignocellulosic counterparts [8, 9]. In general, the *β*-1,3-linkage is an exclusive feature of marine xylans [8]. In green algae like *Caulerpa prolifera*, xylans are described to feature pure *β*-1,3-linkages [10]. So far, all macroalgal xylans described in literature are homoxylans with no additional modifications [11]. The biomass of *P. palmata* has been used for the production of various compounds like methane [12], succinic acid [13], or bioethanol [14].

*P. palmata* consists of around one-third of dietary fibers, while the fraction of soluble fibers (12.2–18.9%) are mainly composed of mixed-linkage xylans sizing from 175 to 400 kDa [15, 16]. These xylans can be used for the production of xylooligosaccharides (XOS) which have gained significant interest due to their numerous applications [17]. Besides their prebiotic effects [17–21], they were shown to have immunomodulatory effects [22, 23], antioxidant effects [24], cancer prevention effects [25, 26] or even direct cytotoxic effects on leukemia cell lines derived from acute lymphoblastic leukemia [27]. Mostly, XOS are produced from terrestrial lignocellulosic biomass, such as corn, wheat straw, rice, rye, and others. Due to their strong interaction with, e.g., cellulose and lignin, the xylans of terrestrial origin are often difficult to extract and mostly need pretreatment of the biomass resulting in more energy- and resource-consuming processes [28–31].

As mentioned above, the non-covalently linked xylans in *P. palmata* are easier to extract than the terrestrial ones. Therefore, the usage of algae biomass for the generation of valuable XOS was extensively studied in the last years. The first approach to utilize *P. palmata* biomass for this purpose has been performed by Yamamoto et al. in 2019 [32]. Prior to the XOS formation, they performed extraction of xylans from the biomass to obtain a fraction with high xylan content (52.2%). Two commercial glycoside hydrolases (GH) were used for the enzymatic hydrolysis of this extract to generate 66.6% of XOS from the xylan present in the extract under optimized conditions of pH 4.5, 50 °C for 24 h. Last year, Fujii et al. [33] investigated two *endo*-xylanases from *Streptomyces thermogriseus* for the XOS production from *P. palmata* xylans. Again the xylans were extracted from the biomass prior to enzymatic hydrolysis. Best results (95.8% conversion) were achieved in a two-step approach with 40 h reaction time at 50–60 °C.

In our study, we present a one-step enzymatic hydrolysis of biomass using the *endo*-1,4-*β*-xylanase FO15_GH10 from *Formosa* sp. Hel3_A1_48 [34], directly utilizing the *P. palmata* biomass without the need for prior polysaccharide extraction, saving valuable time and resources. Based on NMR and MS/MS analyses, we could precisely characterize the XOS products and determine the positions of the mixed linkages. In addition, by employing a second enzyme, FO17_GH43 from *Formosa* sp. Hel3_A1_48, we could develop a process to generate only mixed linked XOS. This could allow a more precise investigation of the impact of *β*-1,3-linkages in bioactivities of XOS in future applications.

## Methods

### Materials

*Palmaria palmata* (dulse) biomass was purchased from Algenladen (Attenweiler, Germany) as dried powder (Article.no: AAAF0664) and leaves (Article.no; AAAF0455). Extraction of xylan from the biomass at 80 °C using dH_2_O was performed as described previously [35]. Xylooligosaccharides and xylans were purchased from Megazyme^®^ with the exception of beechwood xylan which was purchased from Carl Roth^®^. *Caulerpa prolifera* xylan was extracted as previously described [35]. Commercial reference materials for structure elucidation were purchased from TCI (xylobiose, Catalog No. X0067), Biosynth (1,4-β-d-xylotriose, catalog no. OX31985), and Synthose (Floridoside, Catalog No. FL210).

### Recombinant protein production and purification

The genes encoding the enzymes FO15_GH10 and FO17_GH43 were identified in metagenomic studies in 2009 and previously described as members of a putative xylan targeting PUL in *Formosa* sp. Hel3_A1_48 [34]. After removal of the signal peptides, the remaining protein sequences were codon-optimized for expression in *Escherichia coli* and ordered as synthetic genes using the pET28a(+) vector backbone. Protein sequences of FO15_GH10 and FO17_GH43 can be found in Supplementary Table 1. The pET28a(+) plasmids encoding each enzyme (1 ng) were transformed into *E. coli* BL21 (DE3). A single colony was inoculated into TB medium containing kanamycin (50 µg mL^− 1^) and grown overnight. Protein expression was carried out in TB auto-induction media consisting of TB media (Carl Roth^®^) supplemented with 20 × NPS (1x), 50 × 5052 (1x), MgSO_4_ (1 mM) and kanamycin (50 µg mL^− 1^) using baffled flasks with a culture-to-flask volume ratio of 1:5. The optical density at 600 nm (OD₆₀₀) was initially adjusted to 0.1, and the culture was incubated at 37 °C with shaking at 140 rpm until it reached an OD₆₀₀ of 0.6–0.8. The culture was then cooled to the expression temperature of 20 °C and expression was carried out for 16–20 h. Cells were harvested by centrifugation at 4500 × g for 30 min at 4 °C. Cell lysis was performed via ultrasonication on ice (2 × 3 min, 50% power, 50% cycle time), followed by centrifugation at 10,000 × g for 10 min at 4 °C. The resulting supernatant was loaded onto a 5 mL HisTrap^™^ (Cytiva) pre-equilibrated with lysis buffer (50 mM Tris–HCl, pH 8.0, 300 mM NaCl, 10 mM imidazole). Chromatography was carried out using the ÄKTA pure chromatography system at the recommended flow rate. After applying the lysate, the column was washed with 20 column volumes (CV) of wash buffer (30 mM imidazole, 50 mM Tris–HCl, pH 8.0, 300 mM NaCl), and the bound enzyme was eluted using a 5 CV gradient up to 500 mM imidazole. Protein content was monitored at 280 nm. Peak fractions were pooled and desalted using PD-10 desalting columns (GE Healthcare) equilibrated with 50 mM Tris–HCl, pH 8.0, and 25 mM NaCl. The purified enzymes were stored at –20 °C. Repeated freeze–thaw cycles were avoided to preserve enzymatic activity. Protein concentration was determined using Pierce^™^ BCA Protein Assay Kits (Thermo Scientific^™^) with exception of the protein determination in the degradation products as the carbohydrates present in the samples would interfere with the assays. The protein content in those samples was determined using the Bradford-based ROTI^®^Quant (Carl Roth^®^) solution. Different concentrations of xylose were used as standards to ensure no interference with the assay.

### Thin-layer chromatography (TLC)

In TLC experiments, XOS (Megazyme^®^, 2 mg mL^− 1^) were incubated overnight at room temperature with the purified enzymes (25 ug mL^− 1^) in 50 mM Tris–HCl, 100 mM NaCl pH 8.0 at a total volume of 50 μL. A negative control, prepared under identical conditions, omitted the enzyme. As TLC standards, XOS ranging from xylose to xylohexaose (X_1_-_6_) were prepared (1 mg mL^− 1^ each) in the same reaction buffer. The resulting hydrolysates were analyzed according to established procedures [36], using silica gel 60 F_254_ plates and a solvent system composed of 1-butanol, acetic acid, and water (2:1:1, v/v/v). Following separation, the plates were sprayed with a staining solution containing 4 g *α*-diphenylamine, 4 mL aniline, 200 mL acetone, and 30 mL 80% phosphoric acid (v/v). The chromatographic spots were then visualized by heating the plates to temperatures above 100 °C using a heat gun.

### Fluorophore-assisted carbohydrate electrophoresis (FACE)

For separation of the degradation products of enzymatic hydrolysis, the hydrolysates were lyophilized (10 μL) and labeled using the fluorophore 8-aminonaphthalene-1,3,6-trisulfonic acid (ANTS, Invitrogen^™^). Samples were incubated with 4 µL ANTS (0.15 M in 15% acetic acid) and 4 µL NaBH_3_CN (1 M). Due to ANTS strong negative charge, labeled oligosaccharides can be separated solely by size using a 30% acrylamide running gel (250 mM Tris–HCl pH 8.8, 0.1% APS, 0.05% TEMED) and a 10% stacking gel (125 mM Tris–HCl pH 6.8, 0.05% APS, 0.1% TEMED). Labeled samples were mixed with 2 × loading dye (62 mM Tris–HCl pH 6.8, 0.014% bromophenol blue, 10% glycerol). Electrophoresis was performed in running buffer (25 mM Tris, 192 mM glycine) under constant cooling of the chamber in ice at 400 V for about 1 h, until the ANTS front nearly exits the gel. The gels were captured at 312 nm using a UV table.

### 3,5-Dinitrosalicylic acid (DNS) reducing end assay

For the reducing end assay, each sample and standard (20 μL) were mixed with 20 μL DNS reagent (1% DNS, 30% potassium sodium tartrate, 0.4 M NaOH) and incubated at 100 °C for 15 min. Afterwards, 180 µL dH_2_O was added to each sample and 200 µL of the mixture was transferred to a bottom flat microtiter plate. Measurement was performed at 540 nm. A blank of the corresponding background was subtracted from the sample values. For calculation of the XOS concentration, xylose standards (20–1 mM) were prepared and measured analogous. Standards of xylotriose showed similar values. The samples were diluted prior to the assay until the measured values were within the range of the standards concentration. The linear regression equation was used to calculate the equivalents of xylose present in the samples.

### NMR and MS/MS analysis

All NMR spectra were recorded at 297 K in D_2_O on a Bruker AVANCE III HD 600 MHz spectrometer, equipped with a Prodigy N-cryo probe head. Chemical shifts (*δ*) and coupling constants (*J*) were expressed in ppm and Hz, respectively. Before measuring, samples were lyophilized three times from D_2_O to suppress residual –OH peaks in the spectra. All recorded ^1^H spectra are referenced to the residual solvent peak of 4.79 ppm, and ^13^C were referenced via absolute referencing with the corresponding ^1^H-NMR as reference. Initial measurements of the samples showed formation of transient 1-amino sugars, as a result of multiple lyophilizations with residual ammonium carbonate buffer stemming from SEC. These species were already described in earlier studies [37] and could be removed by adjusting the pH of the samples to 4.0–4.5 by addition of small amounts of AcOH-*d4*. A Waters Xevo TQ-S micro Triple Quad MS/MS was used to determine molecular masses and fragmentation patterns of the samples. Mass spectra were recorded via combined fusion method from samples in D_2_O and ACN:H_2_O 1:1 (with 0.1% formic acid). Solvent flow was set to 0.5 mL at a sample co-injection rate of 10 µL min^− 1^.

LC–MS analysis was performed on a Shimadzu LC–MS system equipped with a LC-40DXR solvent delivery module, a DGU-405 degassing unit, a CTO-40C column oven, an SPD-M40 Photo diode array detector, an ELSD-LTIII evaporative light scattering detector, and an LCMS-2050 mass spectrometer. Method gradients were between acetonitrile and water with 0.1% formic acid. Separations were done using a HILIC column, and a 5–95% gradient over 3 min.

### Monosaccharide composition analyses

For determining the monosaccharide composition of the hydrolysates using the *P. palmata* extract and biomass, filtered hydrolysates (< 10 kDa) were used. The samples were dried, weighed, and set to a concentration of exactly 1 mg mL^− 1^. Each sample (100 µL) was mixed with 2 M HCl (100 µL) and incubated at 100 °C for 24 h in pre-combusted glass vials. Samples were analyzed via HPAEC-PAD as described previously using a CarboPac PA10 analytical column (2 × 250 mm) and the Dionex ICS-5000 + system (Thermo Fisher Scientific) with pulsed ampherometric 582 detection (PAD) [38]. Sugars were separated in two steps, an isocratic elution (18 mM NaOH) and a gradient course of two mobile eluent phases (NaOH and CH_3_COONa). Monosaccharide standards (10–1000 μg L^− 1^) were used to identify peaks and quantify the amounts of monosaccharides.

### Large-scale *Palmaria palmata* biomass degradation

To minimize possible microbial contamination, for each reaction, 1 g of *P. palmata* red algae powder was weighed in 50 mL Falcon tubes and initially washed with 20 mL of 70% ethanol for 10 min under shaking at 20 °C and 60 rpm. The ethanol was completely removed after centrifugation at 1000 rpm for 5 min, and the residual ethanol was evaporated in a sterile environment. The same procedure was repeated twice using sterile water. No mass loss was detected in prior tests using 50 mg of the biomass. The enzyme activity was not altered by the washing steps. A final wash was performed with 40 mL of reaction buffer (50 mM Tris–HCl pH 8.0, 100 mM NaCl) for 10 min with shaking at 60 rpm, and the buffer was completely removed. For the reaction, the washed biomass was resuspended in 20 mL reaction buffer containing 100 µg mL^− 1^ ampicillin to prevent growth of bacteria during reaction. The final biomass concentration was, therefore, 50 mg mL^− 1^. Each enzyme was added at 0.2 mg to the respective reactions and incubated at 20 °C and 60 rpm. An additional 0.2 mg of each enzyme was added after 24 h, 48 h, and 72 h to ensure complete xylan degradation. The remaining biomass was removed from the reaction after 96 h by centrifugation for 20 min at 4500 × g and filtration (0.45 µm). To degrade the residual oligo- and polysaccharides, 0.2 mg of the enzymes were added again. After in total of 120 h reaction time, the reaction was centrifuged at 4500 × g for 20 min, filtered through a 10 kDa filter, and frozen at − 20 °C until further use. Time samples of the reaction were taken after 0 h, 4 h, 10 h, 24 h, 48 h, 72 h, 96 h (before and after biomass removal), and 120 h (before and after centrifugation and filtration) centrifuged at 1000 × g for 5 min and inactivated at 80 °C for 10 min to prevent further reaction. Samples were stored at − 20 °C until further processing. All time samples were used for DNS assays and FACE analyses.

### Carbohydrate content determination

To determine the total carbohydrate content present in the dried degradation products of the large-scale (1 g) biomass degradation, the thymol assay was performed based on previously described protocols [39]. Each sample and standard was weighed and exactly set to a concentration of 2 mg mL^− 1^. Thymol reagent (1 mg mL^− 1^ thymol in 98% sulfuric acid) was freshly prepared before every measurement. Samples, standards, and blanks (60 µL) were mixed with the reagent (180 µL) and incubated at 100 °C for 30 min to ensure full hydrolysis of glycosidic bonds. After a short cooldown, samples were mixed well and 100 μL were transferred to a flat-bottom microtiter plate. 100 µL of dH_2_O were added, the plate was shaken well, and the absorbance was measured at 509 nm. As standards, xylose and beechwood xylan (0.1–5 mg mL^− 1^) were prepared. The carbohydrate content of each sample was calculated based on the linear regression equation of the xylose standards. All measurements were carried out in technical triplicates.

## Results

### Identification of xylooligosaccharides (XOS) from *P. palmata* produced by endo-1,4-β-xylanase FO15_GH10 via NMR spectroscopy and MS/MS analyses

As polysaccharide extracts from *P. palmata* are known for their high xylose contents, the red alga can serve as a valuable source for xylans and xylooligosaccharides (XOS). For the initial degradation of polysaccharide extract of the algae, an *endo*-acting enzyme was chosen to investigate its capability to produce XOS from *P. palmata* xylan.

**Fig. 1:**
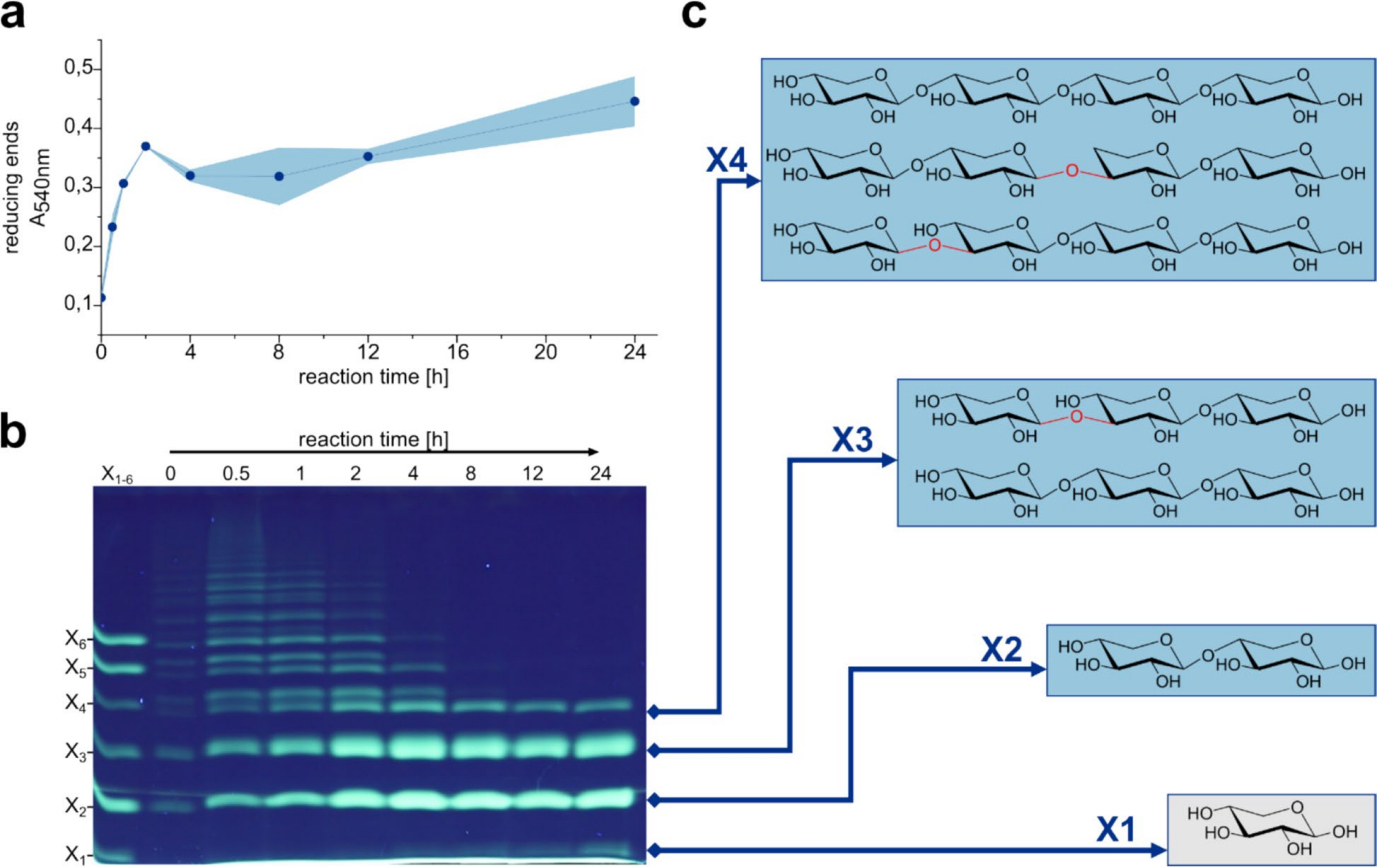
Degradation of *P. palmata* xylan extract by *endo*-1,4-*β*-xylanase FO15_GH10. The formation of reducing ends **a** was monitored during the reaction of FO15_GH10 with xylan extract (5 mg mL^**− **1^) at room temperature and pH 8.0 for 24 h. Standard deviations are shown as a blue shadow. FACE analysis **b** was performed to investigate the product formation over time. After no change in the observed product bands was observed, the reaction was filtered and the products < 10 kDa were purified using size exclusion chromatography. Three product fractions could be identified using NMR and MS/MS. The verified product structures of X2, X3, and X4 are shown **c** in blue boxes. The X1 band at the level of xylose could not be purified (grey box), but since the starting substrate consists almost exclusively of xylose units, it can be assumed that the lowest product band represents xylose. The *β*-1,3-linkage of the XOS is highlighted in red

After identification of the enzyme FO15_GH10 (Fig. S1), a glycoside hydrolase of family GH10 from *Formosa* sp. Hel3_A1_48 [34], the enzyme was produced using recombinant protein expression in *E. coli* BL21 (DE3), followed by His-Tag affinity chromatography to purify the enzyme. We performed initial activity screens of the putative xylanase to identify its ability to act as an *endo*-1,4-*β*-xylanase, accepting a variety of substrates like arabino- and glucuronoxylans, while 1,3-linked xylan like those originating from *Caulerpa prolifera* (CPX) are not targeted by the enzyme (Figs. S2 and S3). FO15_GH10 is also able to degrade smaller XOS like xylohexaose down to xylobiose and xylose (Fig. S4a), while it cannot further degrade xylobiose to xylose (Fig. S4b). Based on the variety of xylans targeted by the enzyme, unsurprisingly FO15_GH10 is also capable of degrading the extracted xylan from *P. palmata* (Fig. 1a). Upon complete degradation, four distinct bands corresponding to degradation products about the size of xylose to xylotetraose can be observed via fluorophore-assisted carbohydrate electrophoresis (FACE) analysis. To further investigate the XOS products, the hydrolysate was filtered (10 kDa MWCO) to remove the enzyme and potentially remaining polysaccharides. The XOS were separated using size exclusion chromatography (SEC) and characterized using NMR spectroscopy and MS/MS analyses.

Initially we aimed to confirm the length of the oligomers suggested by SEC and FACE (Fig. 1b) to further clarify if any non-xylose sugar units were present in the samples. We performed multiple LC–MS runs showing mostly clean peaks and mass distribution for all samples, suggesting successful SEC separation, and fractions of equal oligo-lengths. MS/MS analysis revealed 564 m/z for X4, which corresponds to [M + NH_4_]^+^ of a pentose-tetramer. Present fragments of mass 432 m/z that could correspond to trimeric oligosaccharides could be pinpointed to be a daughter ion of the 564 m/z signal via MS/MS daughter-ion scan with the 432 m/z signal only appearing with the quadrupole filter set to 564 m/z. The same analysis gave 432 m/z for X3, corresponding to a trimeric structure and 300 m/z for X2 indicating a dimer. Structure elucidation was started with commercial reference materials for xylobiose and 1,4-β-d-xylotriose. These standards were fully characterized with ^1^H, ^13^C (DEPTQ), HSQC, COSY, TOCSY and HMBC spectra, and unambiguously all signals were assigned. The full assignments of those two standards are reported in Tables S2 and S3.

After assignment of the standards, we began with the SEC fraction containing the smallest oligos–X2, which turned out to be pure xylobiose, as the arising spectrum matched the one of xylobiose perfectly. With matching NMR and MS data, X2 could be unambiguously assigned as being 1,4-β-d-xylobiose (Fig. S5). Similarly, fraction X3 was identified to contain mostly 1,4-β-d-xylotriose, but also clearly visible signs of different sugars and/or linkages (Fig. S5).

Two species different from the main standard 1,4-linked xylotriose were identified, with the help of band selective 1D-TOCSY experiments, combined with standard 2D experiments. For the extra anomeric proton at 5.14 ppm, an HSQC correlation to a carbon at 98.1 ppm was identified. Selective 1D TOCSY for the signals at 5.14 ppm and 3.90 ppm gave rise to an incomplete carbohydrate pattern, with an axially configured O4 position. Tracing the signals in COSY and HMBC, revealed a six-membered sugar, that had to be galactose. Incomplete magnetization transfer over the whole spin system is common for galactose patterns, as equatorial/equatorial coupling constants of H3 and H5 to H4 are very small. What was left to identify was whether the galactose was glycosylated to any of the xylose units, or if it was maybe bound to the xylose backbone. Besides integrals not allowing for any galactose unit being bound to xylose, HMBC revealed a link towards a glycerol structure. We hypothesize that the molecule could be floridoside (glycerol 2-*α*-d-galactopyranoside) (Fig. S7), a common osmolyte and storage carbohydrate found in red algae [40, 41] which is also known to have immunomodulatory function [42]. Comparison with the reference material revealed a perfect match between the visible signals in X3, as well as exact overlap of the 1D TOCSY traces we recorded (Fig. S8).

For the second component in question (signal at 4.68 ppm, blue marking in Fig. S6), band selective TOCSY together with 2D-COSY analysis revealed a xylose coupling pattern, with an uncommon shift of the anomeric position compared to the main components. HSQC gives a ^13^C shift of the C1 of this xylose at around 103 ppm, thus marking it as clearly glycosylated. Further, assignment of the arising signals from the 1D TOCSY revealed a clearly non-glycosylated O4 position for this sugar unit (see Fig. S9). Next, we aimed to elucidate the glycosylation pattern corresponding to the signal at 4.68 ppm. Looking into the HMBC spectra, a correlation is observable that is distinctly different from any HMBC correlation any of the other anomeric positions show—a carbon at 83.5 ppm. (See Fig. S10, blue marking). The HSQC correlation to that carbon gives a proton shift of 3.62 ppm (Fig. S11). Looking into the COSY spectra again (see Fig. S12) reveals another sugar unit that has very uncommon shifts compared to the standard *β-*1,4-linked xylose-backbone. It consists of a downfield shifted H2 and H3 signal compared to the standard xylotriose, with the H3 matching the glycosidic bond signal found in the HMBC/HSQC earlier, and the H1 signal resides together with the other standard *β-*1,4-linked anomeric signals. We, therefore, concluded that this untypical sugar unit is the second unit being linked *β-*1,4 to the reducing end, but is itself glycosylated at the O3 instead of the O4 position.

The sample content of X3 can, therefore, be estimated to be: 22% floridoside, 17% xylotriose with a 1–3 link towards the non-reducing end, and 61% regular *β-*1,4-d-xylotriose (Fig. S13). These values derive from single NMR analyses and should, therefore, be treated with caution.

Next the SEC fraction X4 was elucidated. We could quickly establish that the general nature of the sample was very similar to the ones already discussed. From LC–MS and MS/MS analysis, we expected a tetrameric structure of pentoses, which was also supported by the ratio of anomeric signals to the other signals in the spectrum. Based on the band height near to the xylotetraose standard (Fig. 1b), we believed xylotetraose to be the most likely structure in the sample. To rule out any other sugar unit but xylose, we scanned the ^1^H spectrum thoroughly via band selective 1D-TOCSYs, but found only familiar coupling patterns, thus letting us conclude that we indeed had a purely xylose-containing sample. Looking into the anomeric region (Fig. S15), we found very similar additional anomeric signals as already seen in X3, that would indicate partly 1,3-linked sugar units. This time however, there were two distinct such sets, instead of only one found in X3. We applied the same reasoning as already in X3, to prove that both signals were indeed of 1,3-linked anomeric nature, and could further show one being a non-reducing end, while the other clearly carrying a glycosylation on O4 (compare trace 3 and 4 from the top in Fig. S14). We concluded that there are 1,3-linkages between unit two and three, and three and four. The contents of this sample are, therefore, three xylotetraoses of different linkages as shown in Fig. S16. The full spectra of all fractions and standards can be found in Figs. S17–S33.

As it is proposed, xylans from *P. palmata* consist of a *β-*1,3/*β-*1,4-mixed linked xylan backbone and most likely do not contain any modifications. The acidic character of the polysaccharide is potentially linked to sulfated or phosphorylated glycoprotein complexes [8]. The structural elucidation of the XOS produced by the degradation of *P. palmata* by FO15_GH10 confirms this assumption. In summary, the upper band at the height of the xylotetraose standard corresponds to a mix of *β-*1,4-linked xylotetraose and two tetraoses consisting of one 1,3-linkage at the second or third linkage position, counting from the reducing end. As we have shown (Fig. S4), FO15_GH10 is capable of degrading *β-*1,4-linked XOS to xylose and xylobiose; therefore, it can be assumed that the degradation of the *P. palmata* xylan was not completed at that point. This is also evident from the presence of *β-*1,4-linked xylotriose in the X3 fraction corresponding to the xylotriose standard, as *β-*1,4-linked xylotriose could be further processed by FO15_GH10 (Fig. S4). Furthermore, the X3 fraction consists of a xylotriose with a *β-*1,3-linkage next to the non-reducing end. As FO15_GH10 is not active on xylobiose, its presence could be confirmed in the X2 fraction, while no *β-*1,3-linked xylobiose could be observed. The X1 fraction could not be isolated but given that the band height is identical to that of the xylose standard, combined with the known release of xylose upon degradation of XOS by FO15_GH10 (Fig. S4), it is reasonable to conclude that the compound is xylose (Fig. 1c).

### FO15_GH10 is capable of producing XOS directly from *P. palmata* biomass

**Fig. 2:**
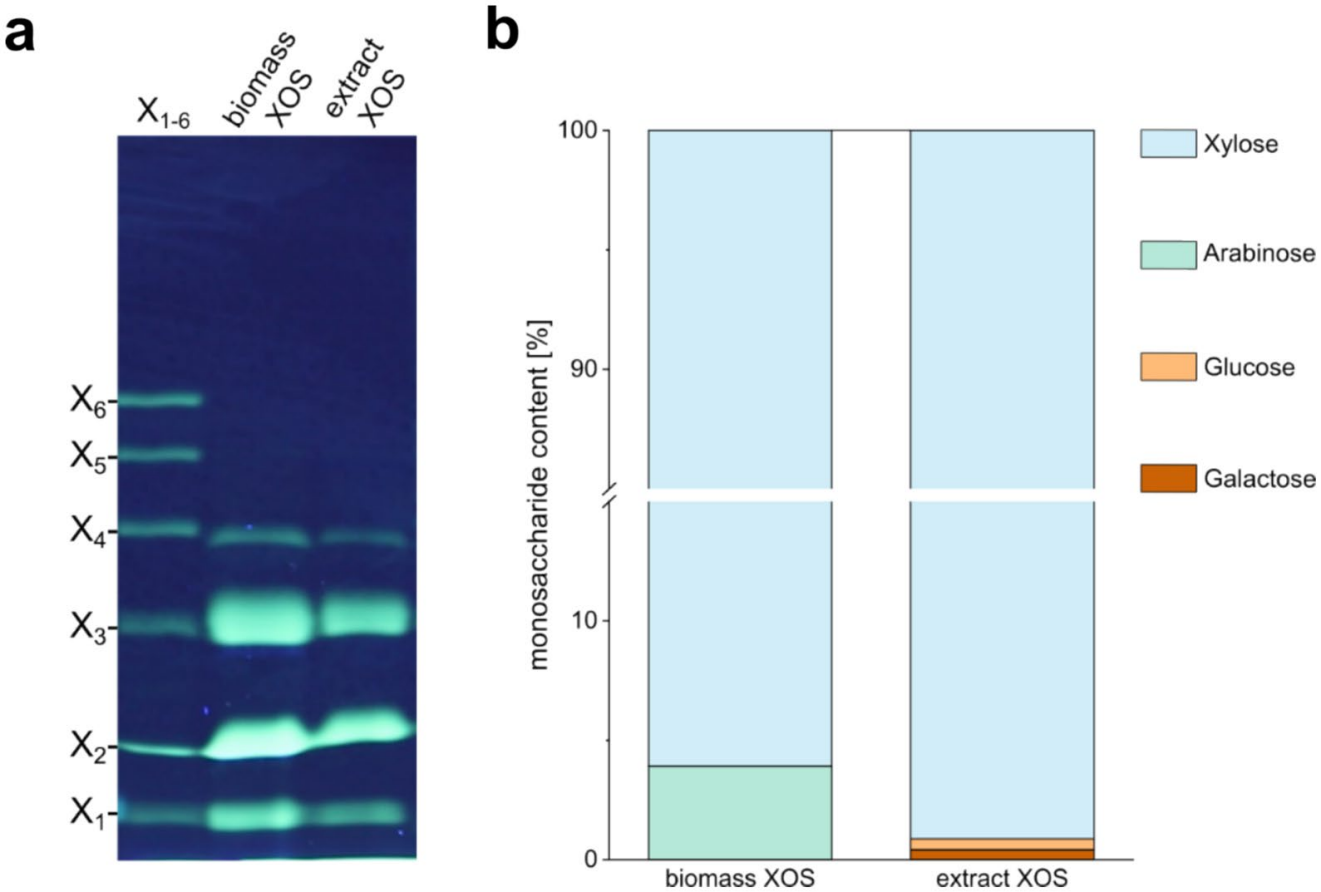
Comparison of XOS from xylan extract and *P. palmata* biomass degradation using FO15_GH10. FACE analyses **a** of the products after degradation of xylan extract (5 mg mL^**− **1^) or *P. palmata* biomass (10 mg mL^**− **1^) at room temperature and pH 8.0 for 24 h. After degradation, the hydrolysates were filtered (10 kDa MWCO) and the monosaccharide contents **b** were determined in a single measurement. No triplicates are presented

As the extraction of polysaccharides from biomass, prior to its utilization, is time and resource consuming, we directly performed enzymatic hydrolysis of the *P. palmata* biomass using FO15_GH10 in a one-pot approach. The only preparation of the biomass consisted of brief washing steps with ethanol, to prevent contamination with microorganisms during the reaction, and water. As indicated by FACE analysis (Fig. 2a), the observed products of the enzymatic hydrolysis of the biomass match the XOS identified after hydrolysis of the *P. palmata* xylan extract. Comparison of the monosaccharide analyses of both, biomass and extract, hydrolysates also shows that the products formed consist almost exclusively of xylose (Fig. 2b). Therefore, it can be assumed that the same XOS as previously characterized (Fig. 1c) are formed upon enzymatic biomass degradation, while no prior extraction steps are needed.

**Fig. 3:**
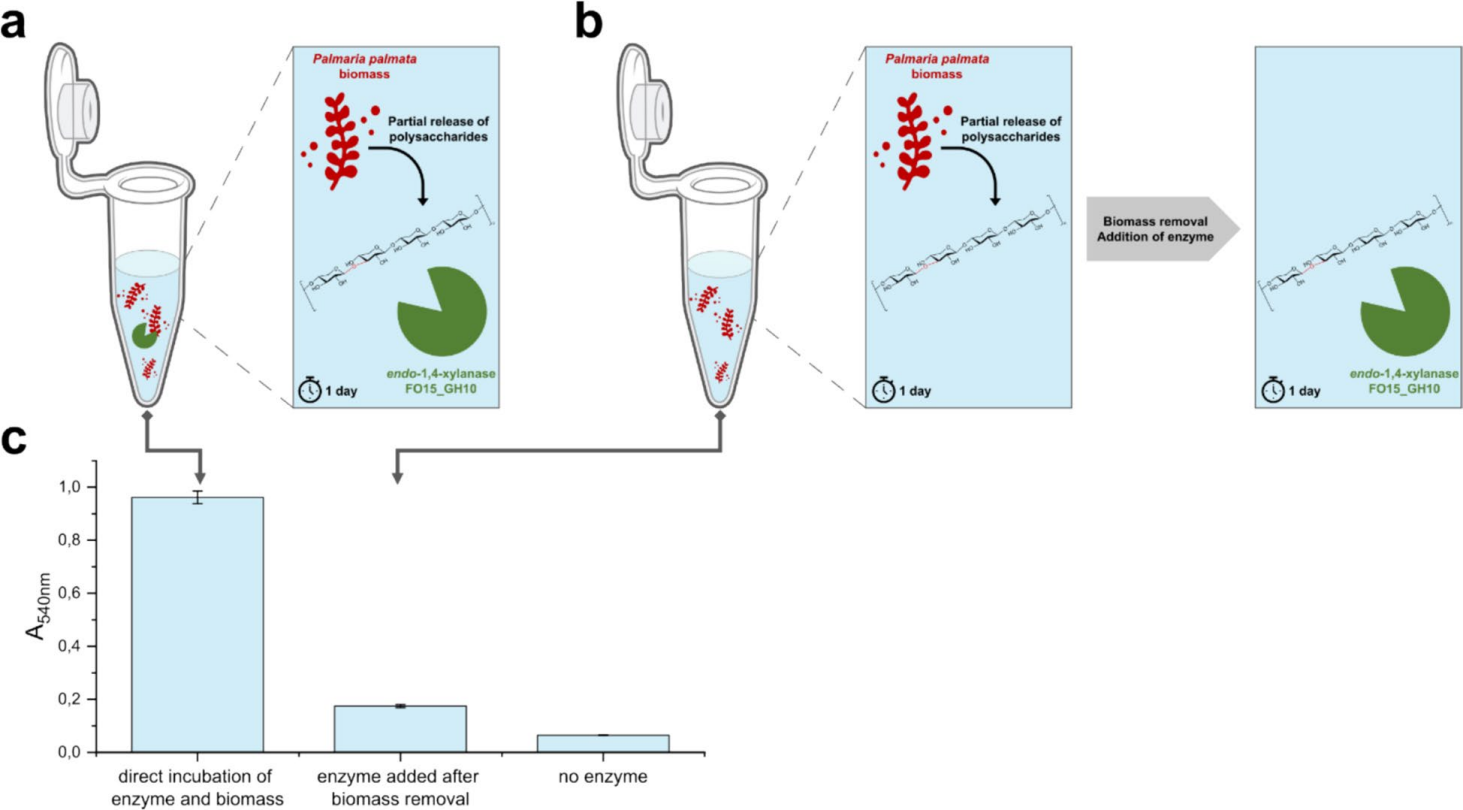
Direct incubation of FO15_GH10 with red algae biomass compared to the degradation of the polysaccharides released by the biomass. After 24 h of incubation of FO15_GH10 (100 μg mL^**− **1^) with the biomass **a** or the prior released polysaccharides, **b** the formation of reducing ends was determined using the DNS-assay. **c**. *P. palmata* powder (25 mg mL^**− **1^) was incubated at pH 8.0 at 20 °C and 750 rpm

In view of the influence of the enzyme on the release of polysaccharide from the red algae biomass, we performed one reaction incubating the enzyme directly with the biomass (Fig. 3a) and one where the algae is only incubated in buffer first releasing polysaccharides to the aqueous environment, then the algae biomass is removed from the reaction and the enzyme is added (Fig. 3b). The comparison of reducing end formation (Fig. 3c) is clearly indicating that the direct contact of enzyme and biomass (Fig. 3a) is enhancing the product formation, therefore facilitating the degradation process substantially. It is likely that the polysaccharides in the algal cell wall are directly targeted by the endolytic activity of the enzyme, which in turn promotes cell lysis and subsequently facilitates the release of additional storage and structure polysaccharides.

### Further use of xylosidase FO17_GH43 enhances xylose production

**Fig. 4:**
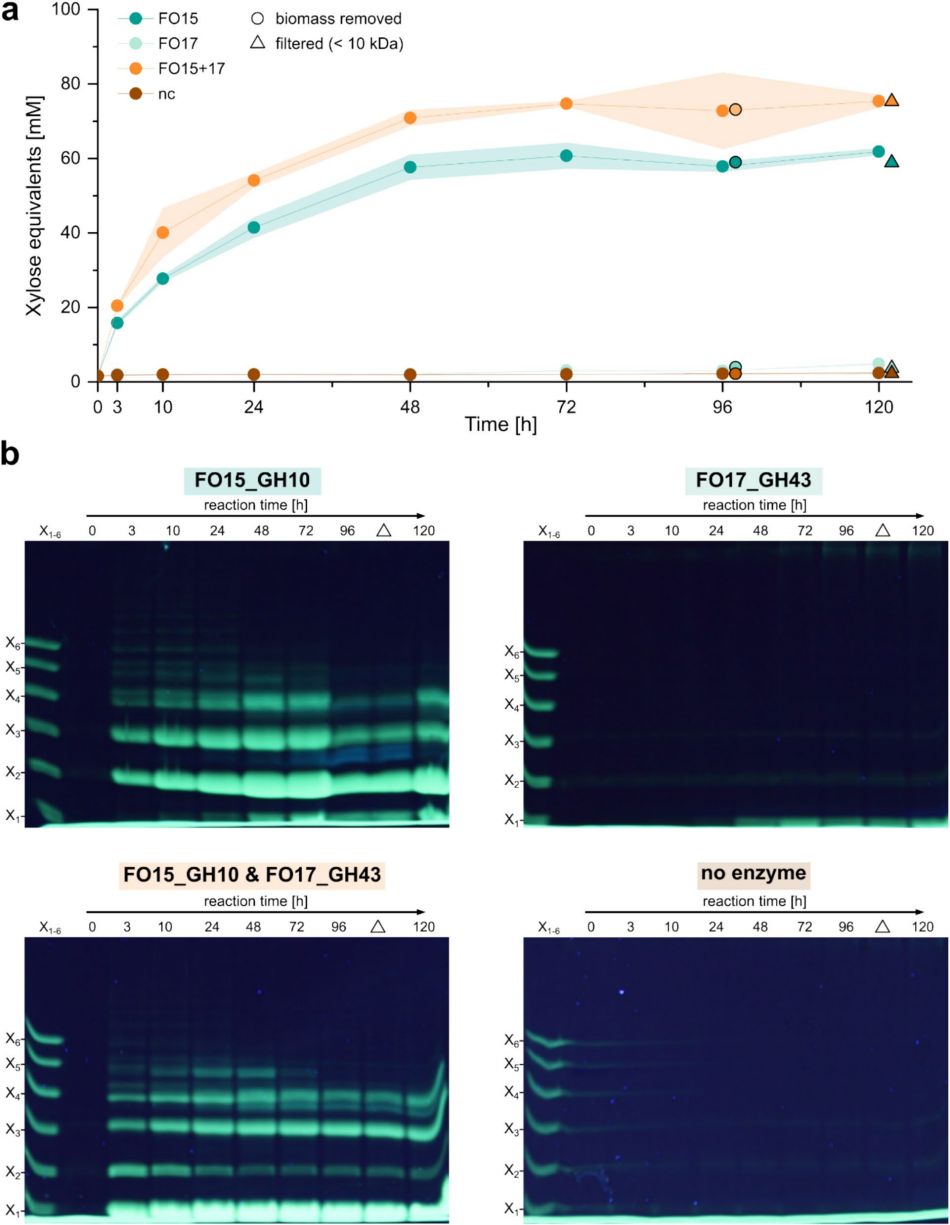
*P. palmata* biomass degradation using FO15_GH10 and FO17_GH43 separately and combined. Xylose equivalents **a** formed upon degradation of biomass with FO15_GH10, FO17_GH43, FO15_GH10, and FO17_GH43 and negative control (no enzyme). FACE analyses of formed products at different time points. **b**. Reactions were performed in 50 mM Tris–HCl, 100 mM NaCl pH 8.0 at room temperature using 1 g biomass (powder) at a total volume of 20 mL (50 mg mL^**− **1^). Enzymes (0.2 mg) were added at 0, 24, 48, and 72 h. After 96 h, the biomass was removed (black circles) and the reaction was continued 1 day to degrade all potential polysaccharide left in the supernatant. After 120 h, the reaction was filtered (10 kDa MWCO, black triangles) to remove the enzymes and other potential molecules deriving from the biomass

Similar to the *endo*-1,4-*β*-xylanase FO15_GH10, the xylosidase activity of FO17_GH43 was investigated using smaller XOS like xylohexaose and xylobiose (Fig. S4). In contrast to FO15_GH10, the *exo*-acting FO17_GH43 is capable of cleaving xylobiose to produce xylose. This can also be seen upon incubation of FO17_GH43 with *P. palmata* biomass (Fig. 4b), where one band at the height of the xylose (X_1_) standard appears and intensifies from 48 h on. However, the amount of xylose released remains low (Fig. 4a). When using both, FO15_GH10 and FO17_GH43, the band pattern shifts in comparison to FO15_GH10 alone (Fig. 4b) and the overall content of xylose equivalents is increased (Fig. 4a). While the X4 and X3 band seem to only lose a bit of intensity, the X2 band vanishes significantly, as FO17_GH43 is capable of degrading *β-*1,4-linked xylobiose which is the only compound of X2 as confirmed via NMR and MS/MS analyses (Fig. 1b). The residual X2 band can potentially be associated with *β-*1,3-linked xylobiose formed upon degradation of mixed XOS by FO17_GH43. As a result of the further degradation of several XOS by FO17_GH43, the intensity of the X1 (xylose) band strongly increases based on the knowledge that FO17_GH43 degrades all *β-*1,4-linked XOS down to xylose (Fig. S4), the all remaining XOS (DP 2–4) have to feature *β-*1,3-/mixed linkages. The use of both enzymes is, therefore, suitable to produce solely mixed linked XOS directly from *P. palmata* biomass.

This experiment further demonstrates that the reaction reaches completion within 48–72 h (Fig. 4a). Consequently, for the large-scale production of XOS, the reaction conditions could likely be optimized to increase efficiency and maximize yield within a reduced timeframe.

To quantify the potential XOS yield after biomass degradation, the filtered reactions were dried, weighed, and contents of carbohydrates and proteins/peptides were measured (Table 1). The yields calculated from the initial 1 g of biomass used for degradation were 17.67 (± 0.16) % when FO15_GH10 was used alone, and 22.60 (± 0.51) % when FO15_GH10 and FO17_GH43 were used together. For FO15_GH10, nearly the entire mass (after filtration) consists of carbohydrates (97.07%), while the approach with two enzymes yielded lower amounts of carbohydrate (75.39%) but higher amounts of xylose. The main components of the approach using FO17_GH43 and no enzyme remain elusive, as the carbohydrate and protein contents were rather low. As the investigated samples underwent no further purification besides filtration, it is hard to determine the components. It is likely that they potentially derive from the *P. palmata* cells itself. For further application of the generated XOS, a suitable purification protocol should be established to purify the oligosaccharides from potential contaminants. It is nonetheless remarkable that such substantial yields of XOS can be obtained from pure biomass using only one (or two) enzymes in a one-pot approach.

**Table 1:** Determined contents and yields after the degradation of 1 g *P. palmata* biomass using FO15_GH10 and FO17_GH43 separately and combined. Carbohydrate content was determined using the thymol assay, while protein concentration was determined using the Bradford assay

	FO15_GH10	FO17_GH43	FO15_GH10 and FO17_GH43	No enzyme
Mass yield [mg]	329	180	331	144
Carbohydrate content [%]	97.07 ± 7.19	10.07 ± 4.33	75.39 ± 8.64	6.82 ± 5.63
Protein content [%]	n.d	0.48 ± 0.08	n.d	0.17 ± 0.08
Xylose eq. [mM]	58.86 ± 0.54	3.69 ± 0.07	75.28 ± 1.71	2.27 ± 0.13
Xylose eq. [mg]	176.73 ± 1.61	11.07 ± 0.22	226.04 ± 5.14	6.83 ± 0.39
yield* [% (w/w)]	17.67 ± 0.16	1.11 ± 0.02	22.60 ± 0.51	0.68 ± 0.04

*The yield was calculated based on the xylose equivalents measured using DNS reducing end assay. As the exact composition of the different XOS is not known, these values are only approximations; n.d.: not detectable

## Discussion

The red algae *Palmaria palmata* serves as an important source for xylans and XOS as the cell wall polysaccharide is easily accessible [8, 9]. Previous studies of enzymatic hydrolysis of *P. palmata* xylan rely on the prior extraction of the polysaccharides from the red algae [32, 33]. Yamamoto et al. were able to extract a xylan-rich fraction (52% xylan) accounting for 13.8% of the dried dulse powder used for the process. Taking into account that 66.6% of this fraction was further processed to XOS, this results in a final XOS yield of 9.19% based on the initial biomass used for extraction [32]. The recent study by Fuji et al. using two endo-xylanases for XOS production also described enzymatic hydrolysis after extraction of xylan from the biomass. Since no yield was reported after extraction, it is not possible to calculate the final yield based on the initially used biomass.

Our approach of directly processing the dried *P. palmata* biomass with FO15_GH10 resulted in approx. 17.67% XOS yield (22.6% for FO15_GH10 and FO17_GH43). Based on the fact that *P. palmata* consists of about 33% total dietary fibers and approx. 12.2–18.9% soluble fibers, including the mixed linked xylans [15], these yields obtained are exceptionally high. The DP of our products is rather low (DP 2–4), which is favorable for the prebiotic benefits of XOS as it was observed that the effects increase with low DP [43, 44]. However, if larger XOS should be preferred, it is likely that other endo-acting xylanases, which can produce higher DP XOS, can be used for the direct biomass utilization as well enable a broad application in various industries.

Nevertheless, it must be taken into account that these products have not yet been purified and that the XOS deriving from biomass conversion are likely more heavily contaminated than those obtained from previously extracted polysaccharides, as various components from the *P. palmata* cells, like peptides, small carbohydrates or other metabolites can still be present after the removal of larger molecules over 10 kDa. Therefore, a loss in yield should be expected during subsequent purification steps required to isolate the XOS.

Similar biomass utilization of untreated kelp biomass using a laminarinase and alginate lyase resulted in a mixture of sugars, usable for microbial fermentation [45]. The hydrolysates obtained by our approach can as well be used for the application in fermentation, where no further purification of the products would be required. For the use of the XOS, purification steps can be employed as shown in several studies [17]. Techniques like ultrafiltration and precipitation can be used to obtain fraction with similar DP and degree of substitution from enzymatically degraded arabinoxylans [46] or mechanically extracted soybean meal [47]. Many other membrane-based techniques can be used for a variety of oligosaccharides as accurately summarized by Pinelo et al. [48] and Wen et al. [49].

To obtain high-purity XOS, e.g., for the use as prebiotics or in the production of pharmaceuticals, chromatographic separation like ion exchange for charged products or SEC, like exemplified in our study, can be performed. As we did not determine what kind of impurities can be found in the hydrolysis products, further research is needed to establish suitable purification procedures of XOS for their application in the food and pharmaceutical industry.

## Conclusion

The use of biomass for the production of valuable bioactive compounds has been of great interest to biotechnology for several decades. In this context, processes are continuously being optimized to establish efficient production of, e.g., bioethanol, fine chemicals or pharmaceuticals. The red alga *Palmaria palmata* contains high amounts of xylans in its cell walls. As these polysaccharides are more readily accessible compared to terrestrial xylans, the algae biomass serves as a valuable source XOS with their various properties like prebiotic [17–21], immunomodulatory [22, 23], antioxidant [24], or cancer prevention effects [25–27].

In our study, we could establish a simple one-step enzymatic hydrolysis approach of *P. palmata* biomass, without the need of prior extraction processes saving valuable resources. This approach not only represents the first described method for directly producing XOS from marine biomass via enzymatic hydrolysis, but also achieved higher XOS to biomass yields (17.6% and 22.6%) than previous investigations which included an extraction step before enzymatic hydrolysis.

## Supplementary Information

Additional file 1.

## Data Availability

Data is provided within the manuscript or supplementary information files.

## Abbreviations

DP: Degree of polymerization
FACE: Fluorophore-assisted carbohydrate electrophoresis
GH: Glycoside hydrolase
Nc: Negative control
TLC: Thin layer chromatography
XOS: Xylooligosaccharides
