# Supplementary material for: Producing mixed linked xylooligosaccharides from red algae biomass through single-step enzymatic hydrolysis
Source: Biotechnol Biofuels Bioprod. 2025 Aug 5;18:87. doi: 10.1186/s13068-025-02686-7 (PMC12323150; doi:10.1186/s13068-025-02686-7)
Supplement: Supplementary file 1 — Additional file 1. [file 13068_2025_2686_MOESM1_ESM.pdf]

Supporting Tables

Table S1. Protein sequences of recombinant expressed FO15_GH10 and FO17_GH43.

**FO15_GH10**	MGSSHHHHHHSSGLVPRGSHMCASKIVSESSSKLSLGTHFKDLFYLGAAINENTILGLDPKSATIVNSEYNTI<br>SPENSLKWMFIQPSPNKFNFKAADKYVEMGLKNNMYIVGHALVWHNQLADFMQNLENSAETRAHVENHIN<br>TLVSRYKGKIDAWDVVNEAFEEDGSLRASVFYKNMGKNYIEEVFRKTEKVDPDVDLIYNDYNLYKPKKRAA<br>VLEMVKKFKANGTKINGVGVQAHWDLKSPSIEEIEQIILDVHAAGVYVSFTELDISVLPNPWEMVGAEVTQN<br>FSQFEGDPKMNPYPNGLPDNIQEKLAKRYQEIFNVFVKHSDKINRVTFWGVMDKHSWLNDWPIKGRTNYP<br>LLFDRNYNPKPAYKSVLEVNTDQQN
**FO17_GH43**	MGSSHHHHHHSSGLVPRGSHMPEDNIDHIDFKALNKKALSAPLVTHIYTADPSAHYFNGKIYIYPSHDIDAG<br>EAFDDLGSHFAMEDYHVISMDNIDSKAVDNGVALHVDDVPWAKQQMWAPDANEKDGKFYLFFPAKDYEGI<br>FRIGVAISDSPTGPFKAEPKAIEGSFSIDPAVFKDDDGAYYMYFGGLWGGQLQRWRTGVFNADQPESPTAF<br>LPADDEPALLPLVAKMSEDLLAFGETPKALEILDENGDLLLSGDNDRRFFEAAWLHKHNGKYYFSYSTGDT<br>HFICYAIGDSPYGPFIYTGRILNPVVGWTSHHSVCEVEGKTYLFYHDSSLSKGITHLRSVKVAELKYRQDGTII<br>TLNPY

Table S2. Assignments of commercial reference material xylobiose (TCI, catalog no. X0067) in D_2O (See Figure S17 and Figure S18 for full spectra)

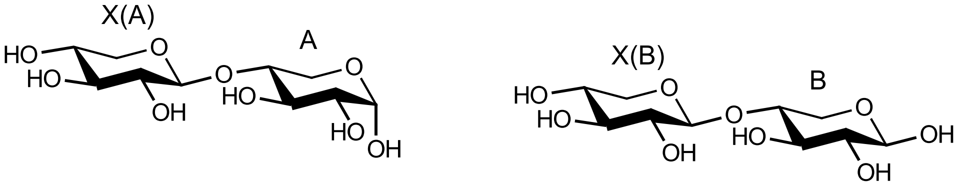

	^1H (in ppm)		^{13}C (in ppm)
A1	5.19	d, J = 3.7 Hz	94.7
A2	3.55	dd, J = 9.2, 3.8 Hz	72.1
A3	3.80 – 3.72	m	73.6
A4	3.80 – 3.72	m	79.3
A5	a: 3.84 – 3.80	m	61.5
	b: 3.80 – 3.72	m	
B1	4.59	d, J = 7.9 Hz	99.2
B2	3.28 – 3.23	m	76.7
B3	3.55	t, J = 9.25 Hz	76.6
B4	3.80 – 3.72	m	79.1
B5	a: 4.06	dd, J = 11.7, 5.4 Hz	65.7
	b: 3.38	dd, J = 11.62, 10.7 Hz	
X1(A)	4.46	d, J = 7.8 Hz	104.54
X1(B)	4.46	d, J = 7.8 Hz	104.55
X2	3.28 – 3.23	m	75.5
X3(A)	3.43	t, J = 9.3 Hz	78.28
X3(B)	3.43	t, J = 9.3 Hz	78.30
X4	3.63l	m	71.9
X5	a: 3.97	dd, J = 9.2, 5.5 Hz	67.9
	b: 3.31	dd, J = 10.8, 0.8 Hz	

Table S3. Assignments of commercial reference material 1,4-β-D-xylotriose (Biosynth, catalog no. X31985) in D_2O (See Figure S19 and Figure S19 for full spectra)

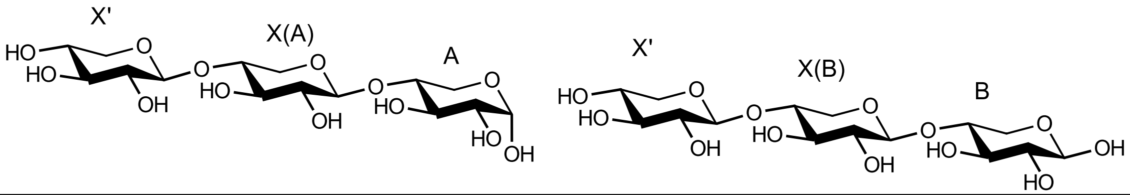

	^1H (in ppm)		^{13}C (in ppm)
A1	5.19	d, J = 3.7 Hz	94.7
A2	3.58 – 3.52	m	74.1
A3	3.77 - 3.72	m	73.6
A4	3.77 - 3.72	m	79.3
A5	a: 3.83 – 3.79	m	61.5
	b: 3.77 - 3.72	m	
B1	4.59	d, J = 7.9 Hz	99.2
B2	3.28 – 3.23	m	76.7
B3	3.58 – 3.52	m	76.6
B4	3.80 – 3.76	m	79.06 (amb. with X4)
B5	a: 4.06	dd, J = 11.7, 5.4 Hz	65.6
	b: 3.38	d, J = 11.3 Hz	
X1(A)	4.48	d, J = 7.7 Hz	104.4
X1(B)	4.48	d, J = 7.8 Hz	
X2	3.32 – 3.27	m	75.4
X3(A)	3.58 – 3.52	m	76.3
X3(B)	3.58 – 3.52	m	76.4
X4	3.80 – 3.76	m	79.08 (amb. with B4)
X5	a: 4.11	dd, J = 11.8, 5.3 Hz	65.6
	b: 3.38	d, J = 11.3 Hz	
X1'	4.46	d, J = 8.0 Hz	104.5
X2'	3.28 – 3.23	m	75.5
X3'	3.43	t, J = 9.3 Hz	78.3
X4'	3.63	td, J = 9.5, 5.5 Hz	71.9
X5'	a: 3.97	dd, J = 11.6, 5.5 Hz	67.9
	b: 3.31	app. t, J = 11.1 Hz	

Supporting Figures

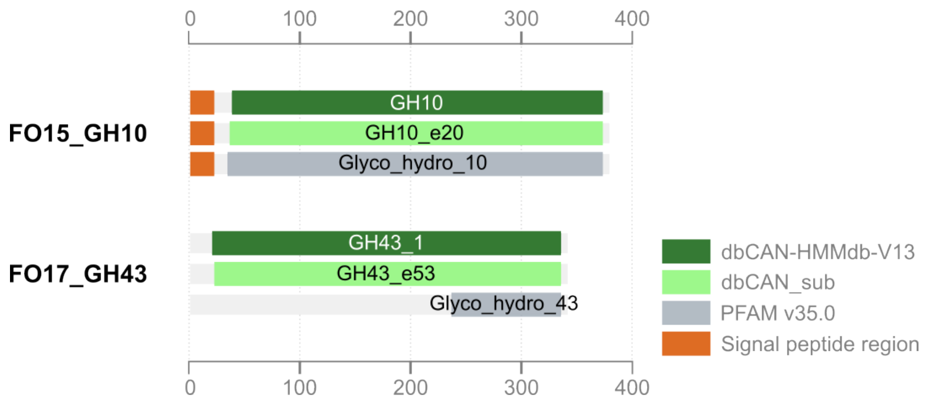

Figure S1. Annotations of the protein sequences of FO15_GH10 and FO17_GH43 based on dbCAN-Hmmdb_v12, dbCAN_sub and PFAM.

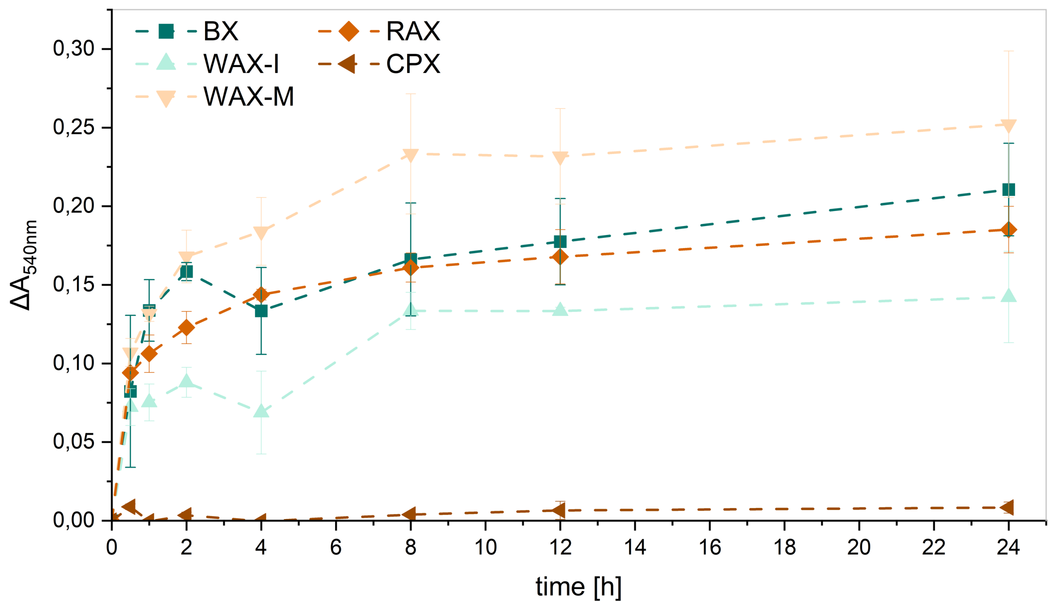

Figure S2. Formation of reducing ends during the degradation of xylan polysaccharides by FO15_GH10. The glucuronoxylan from beechwood (BX), arabinoxylans from rye (RAX) and wheat (insoluble: WAX-I, medium viscosity: WAX-M) and 1,3-linked xylan extracted from *Caulerpa prolifera* (CPX) were tested as substrates from the *endo*-1,4-xylanase FO15_GH10. Reactions were performed using 2 mg mL^{-1} polysaccharide and 25 µg mL^{-1} of FO15_GH10 in 50 mM Tris HCl pH 8.0, 100 mM NaCl pH 8.0 at room temperature for 24 h. Samples were used for DNS reducing end assays. The change in absorbance (ΔA_{540nm}) is linear to reducing ends formed upon xylan degradation. All measurements were carried out in technical triplicates. Mean values and standard deviations are shown.

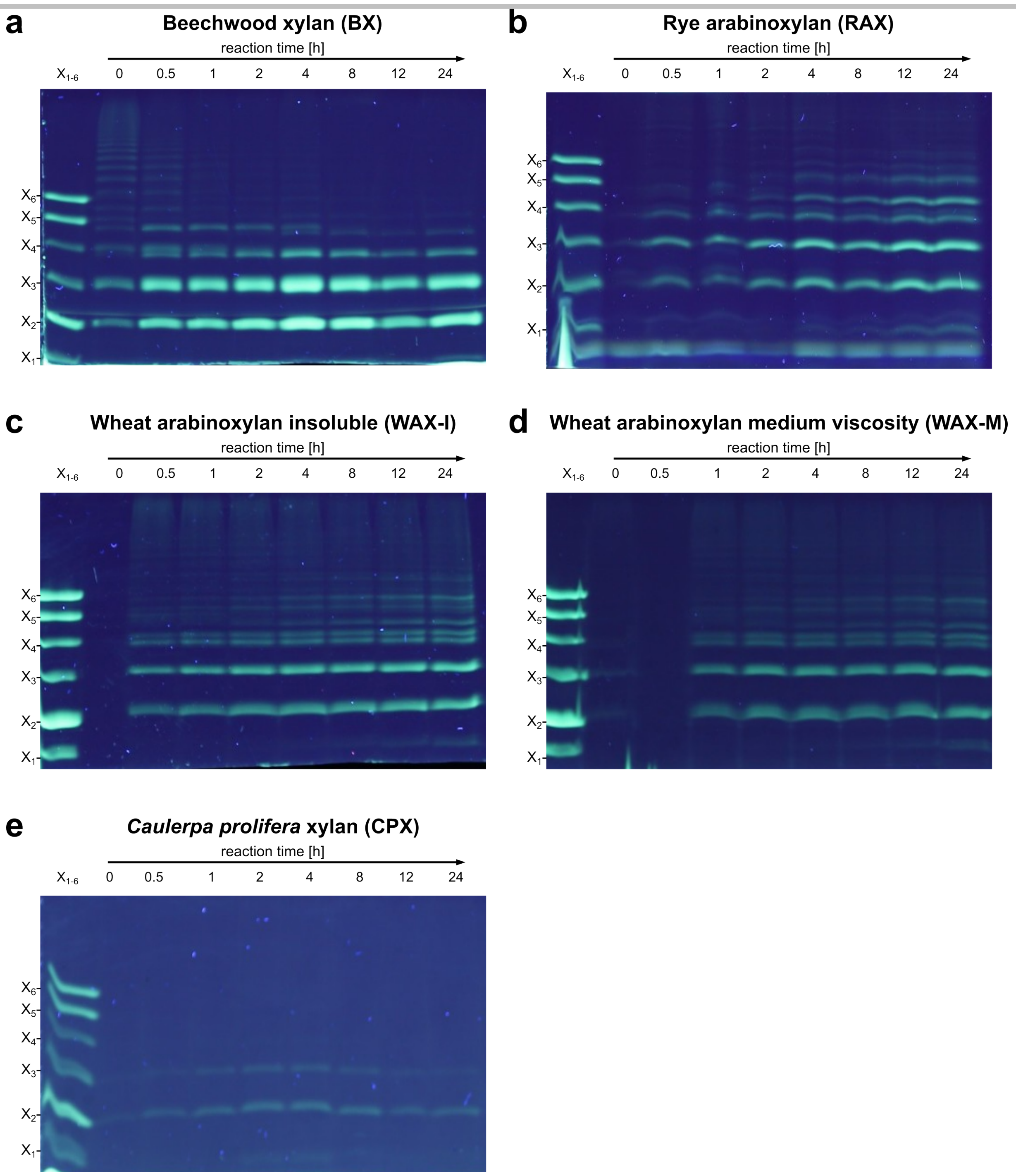

Figure S3. FACE analyses of degradation products of xylan polysaccharides upon degradation by FO15_GH10. The glucuronoxylan from beechwood (**a**), arabinoxylans from rye (**b**) and wheat (**c** and **d**) and 1,3-linked xylan from *Caulerpa prolifera* (**e**) were tested as substrates from the *endo*-1,4-xylanase FO15_GH10. Reactions were performed using 2 mg mL^{-1} polysaccharide and 25 ug mL^{-1} of FO15_GH10 in 50 mM Tris HCl pH 8.0, 100 mM NaCl pH 8.0 at room temperature for 24 h. XOS ranging from xylose to xylohexaose (X_{1-6}) were used as references for the FACE analyses.

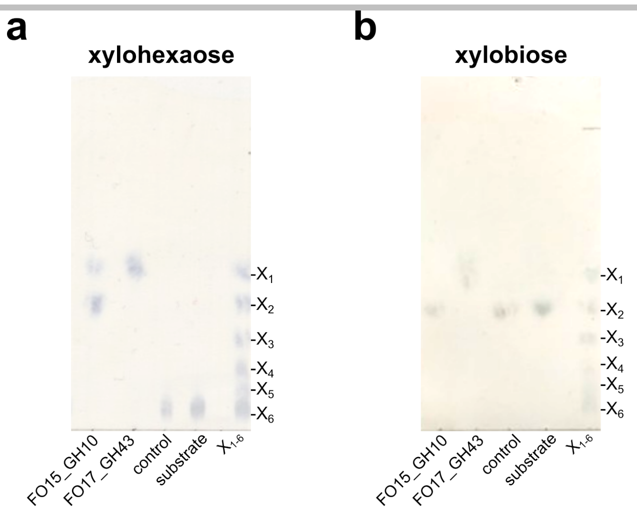

Figure S4. TLC analyses of degradation products of small XOS upon degradation by FO15_GH10 and FO17_GH43. Reactions were performed using 2 mg mL^{-1} substrate and 25 ug mL^{-1} of the respective enzyme in 50 mM Tris-HCl pH 8.0, 100 mM NaCl pH 8.0 at room temperature for 24 h. As a control the substrate was incubated without enzyme. Substrate and XOS ranging from xylose to xylohexaose were used as references for the TLC analyses.

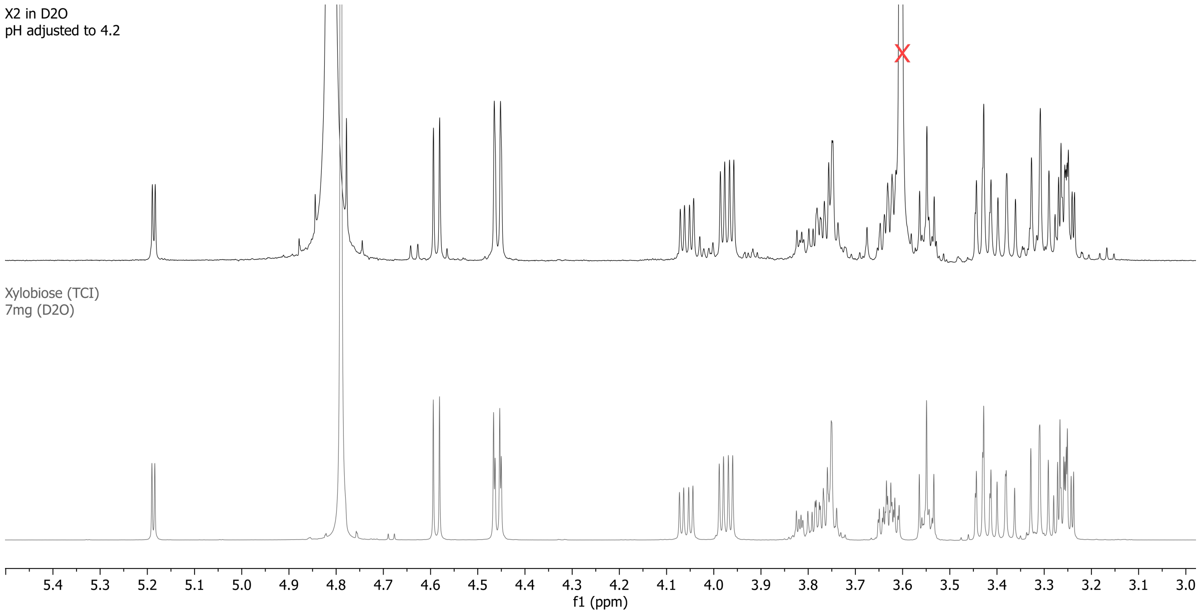

Figure S5. ^{1}H (600 MHz) spectra comparison between SEC fraction X2 (top) and commercial xylobiose (bottom). The red X is marking a signal which does not correspond to the component in the sample.

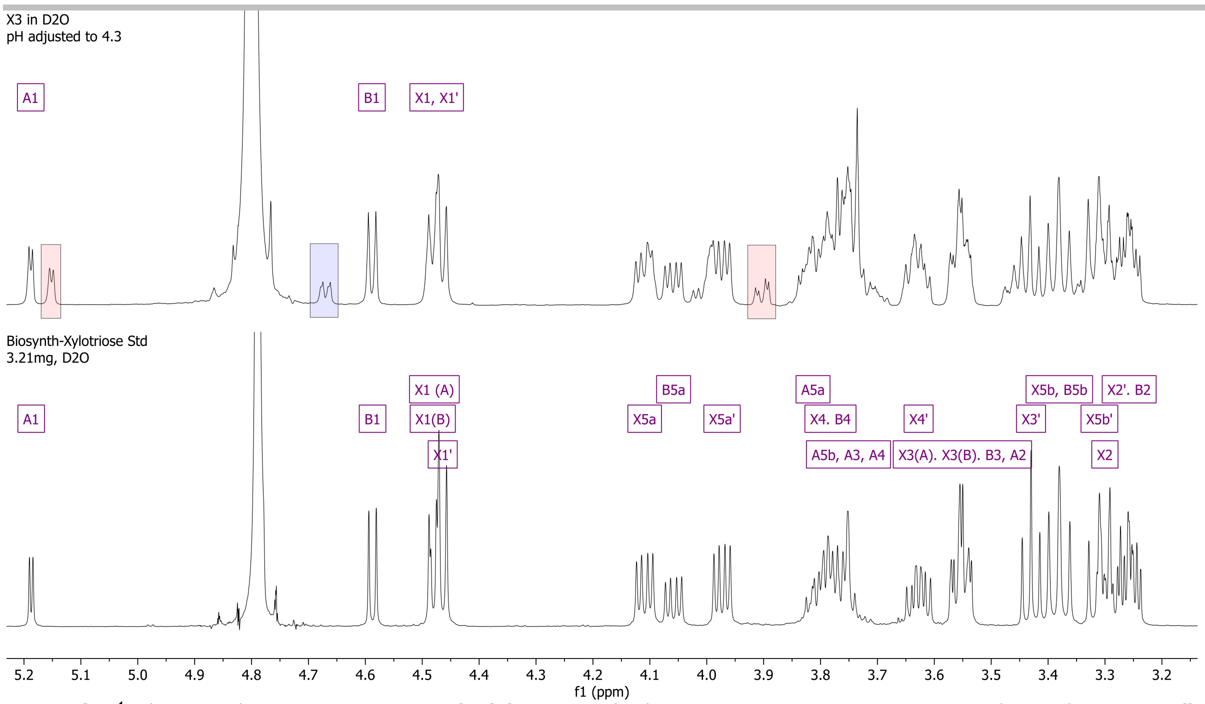

Figure S6. ^{1}H (600 MHz) comparison between SEC fraction X3 (top) and commercial xylotriose standard (bottom). Notable differences between the spectra are colored in blue and red. Signals corresponding to floridoside are colored in red and signals corresponding to the uncoupled O4 position of the 1,3-linkage are colored in blue.

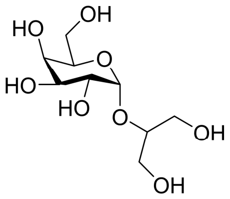

Figure S7. Structure of floridoside.

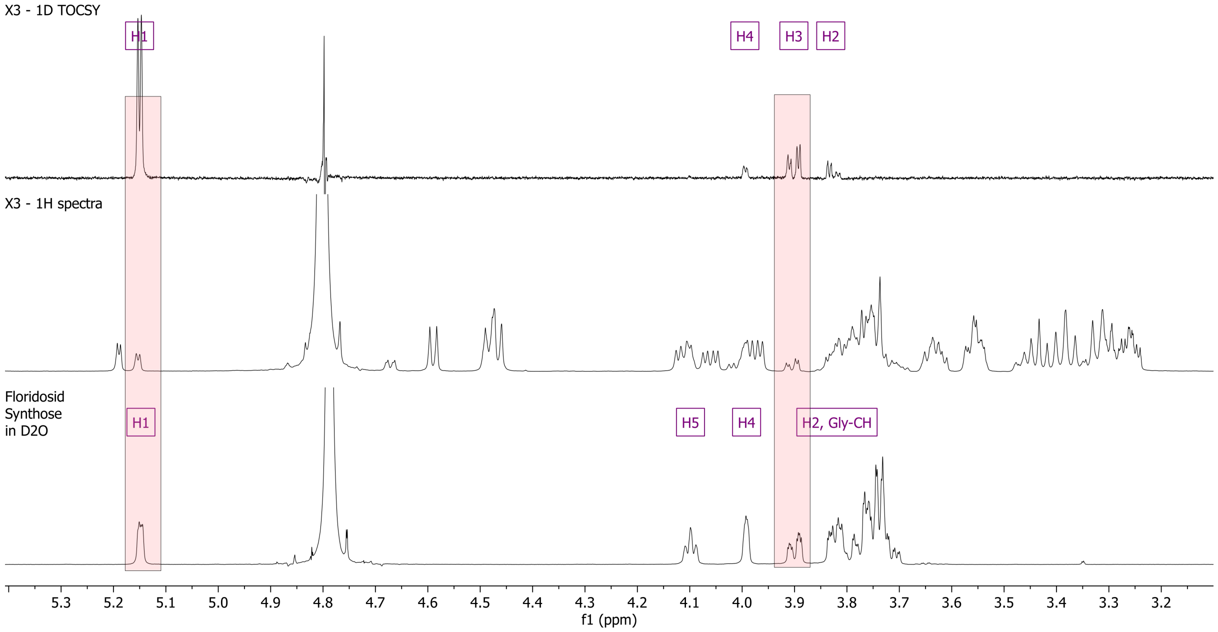

Figure S8. ^{1}H (600 MHz) comparison between SEC fraction X3 (middle), commercial floridoside standard (bottom) and band selective 1D-TOCSY spectra (excitation at 5.14 ppm)

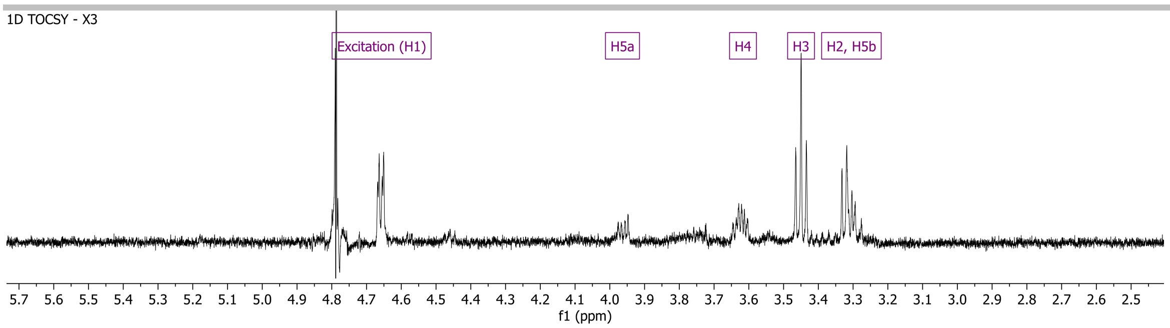

Figure S9. Band selective 1D-TOCSY spectrum of SEC fraction X3. Excitation at the signal at 4.67 ppm, reveals a full xylose pattern, with an uncommon shift for H1.

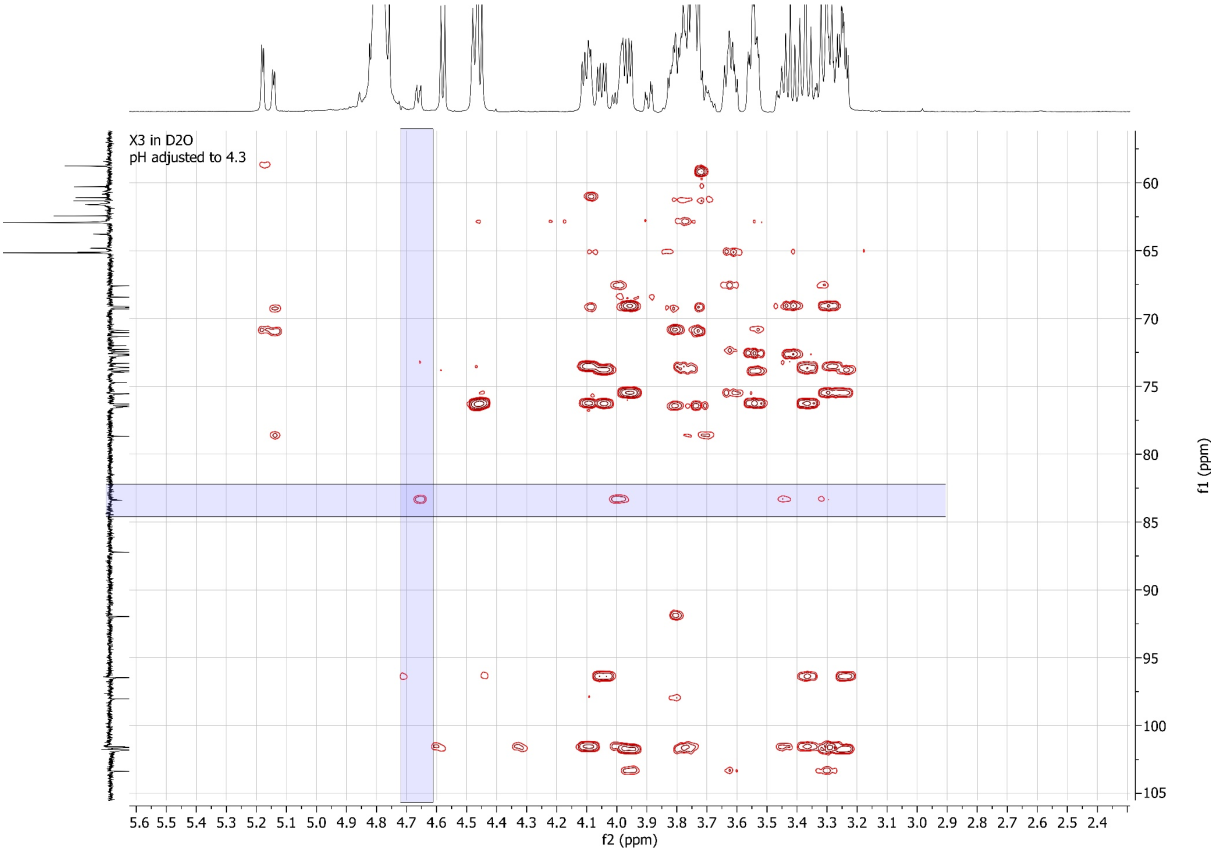

Figure S10. HMBC spectra of X3. Highlighted in blue, are relevant correlations.

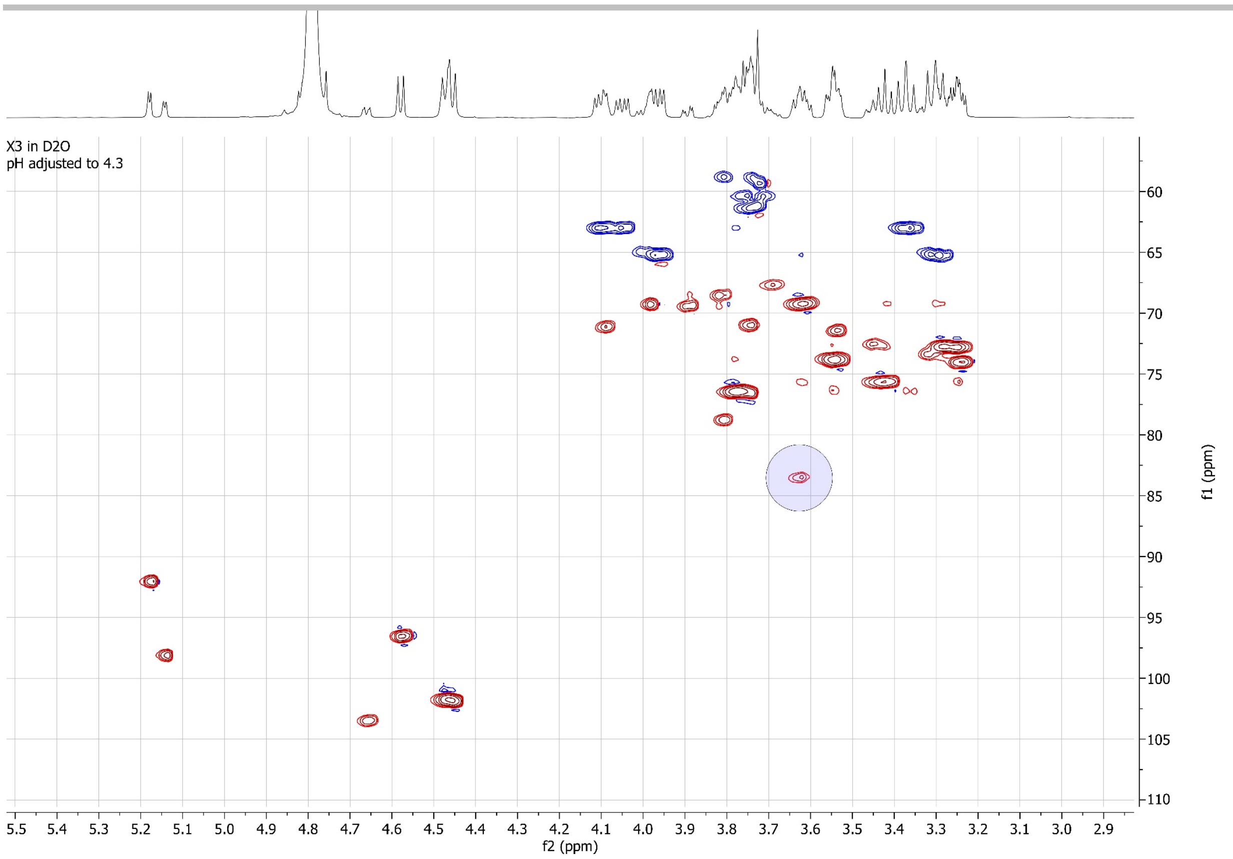

Figure S11. HSQC spectra of X3. Highlighted in blue is the correlation matching at 83.5 ppm.

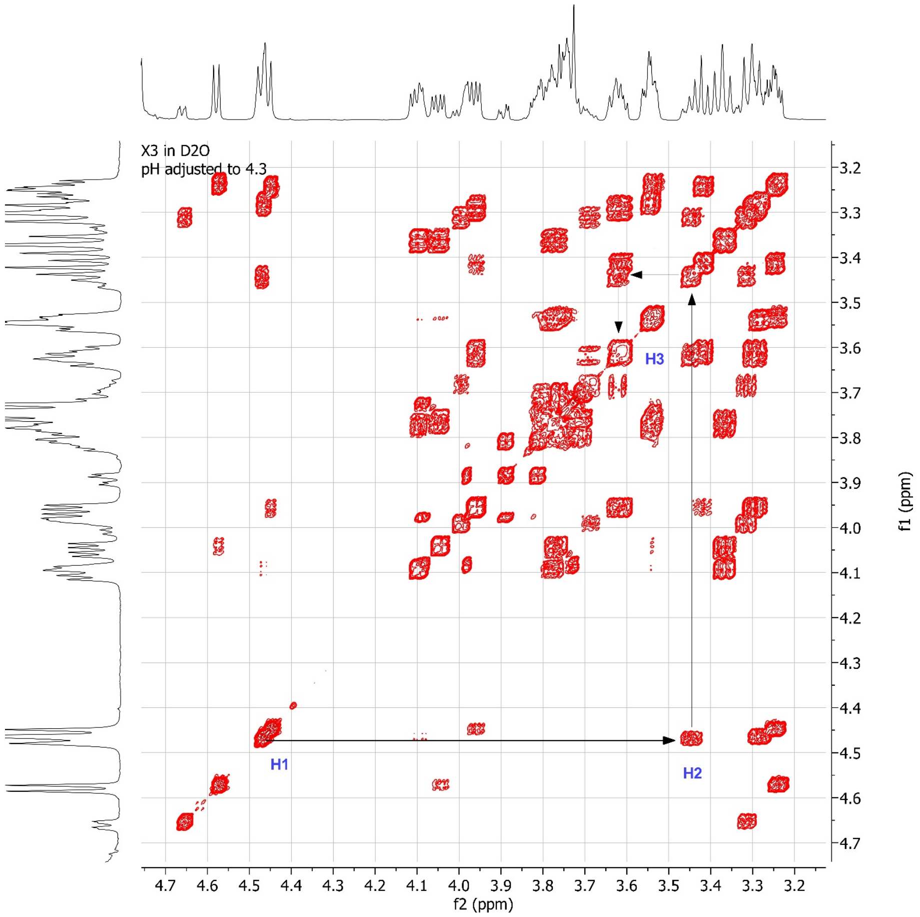

Figure S12. COSY spectra of X3.Highlighted (arrows) are the relevant correlations, that show the sugar unit, that is glycosylated at O3.

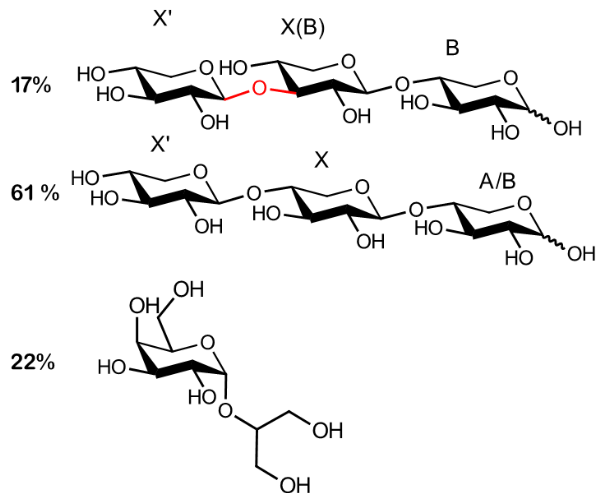

Figure S13. Contents of SEC Fraction X3

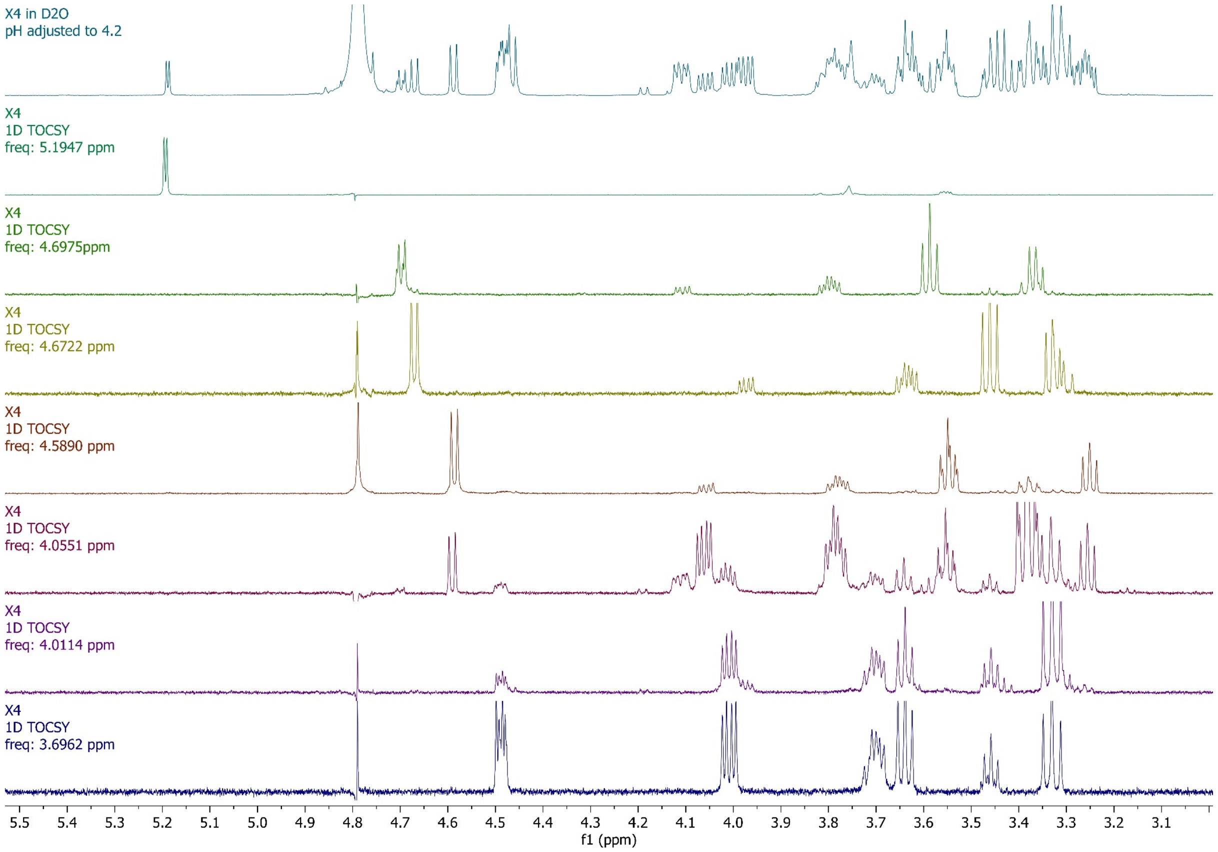

Figure S14. Stackplot of ^{1}H NMR of SEC fraction X4 (top), together with multiple band selective 1D-TOCSY experiments.

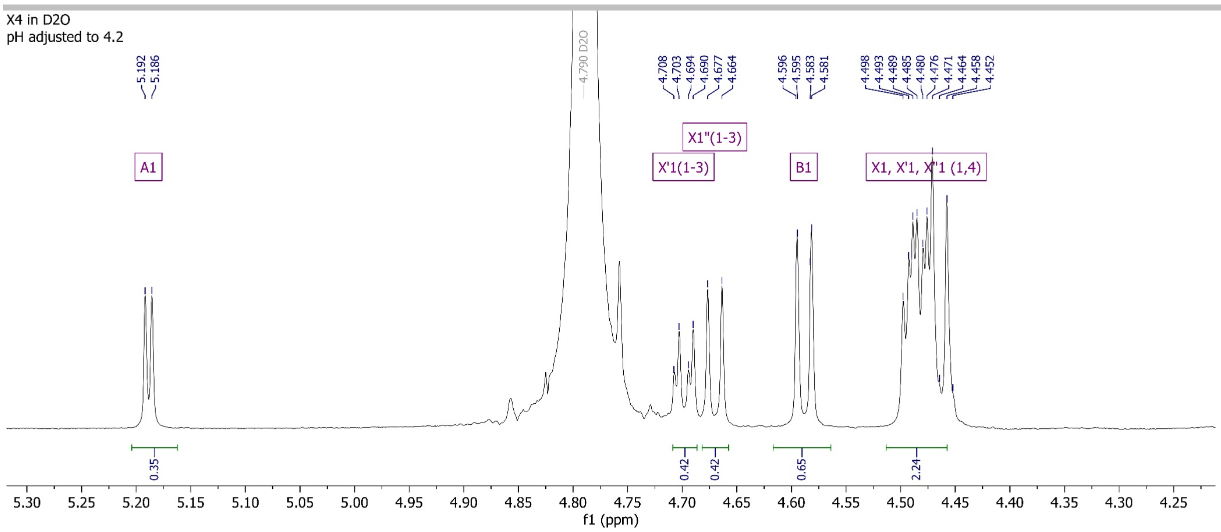

Figure S15. 1H (600 MHz) spectra of SEC fraction X4, zoom into the anomeric region, clearly showing 1,3-linkages.

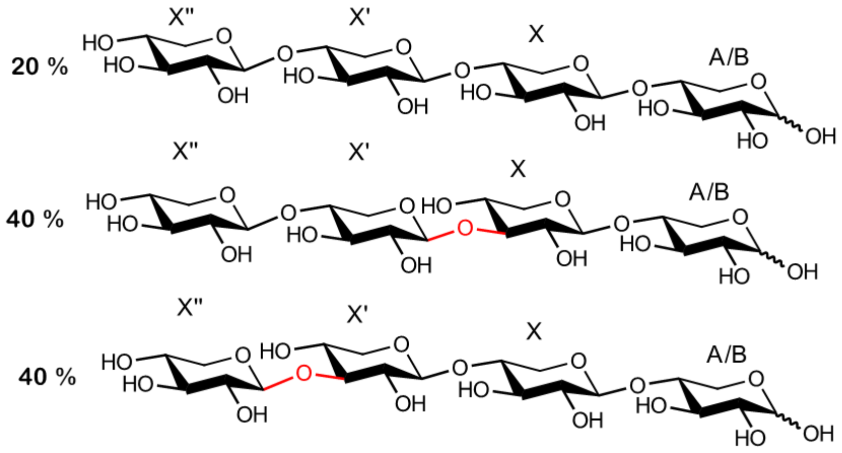

Figure S16. Contents of SEC Fraction X3

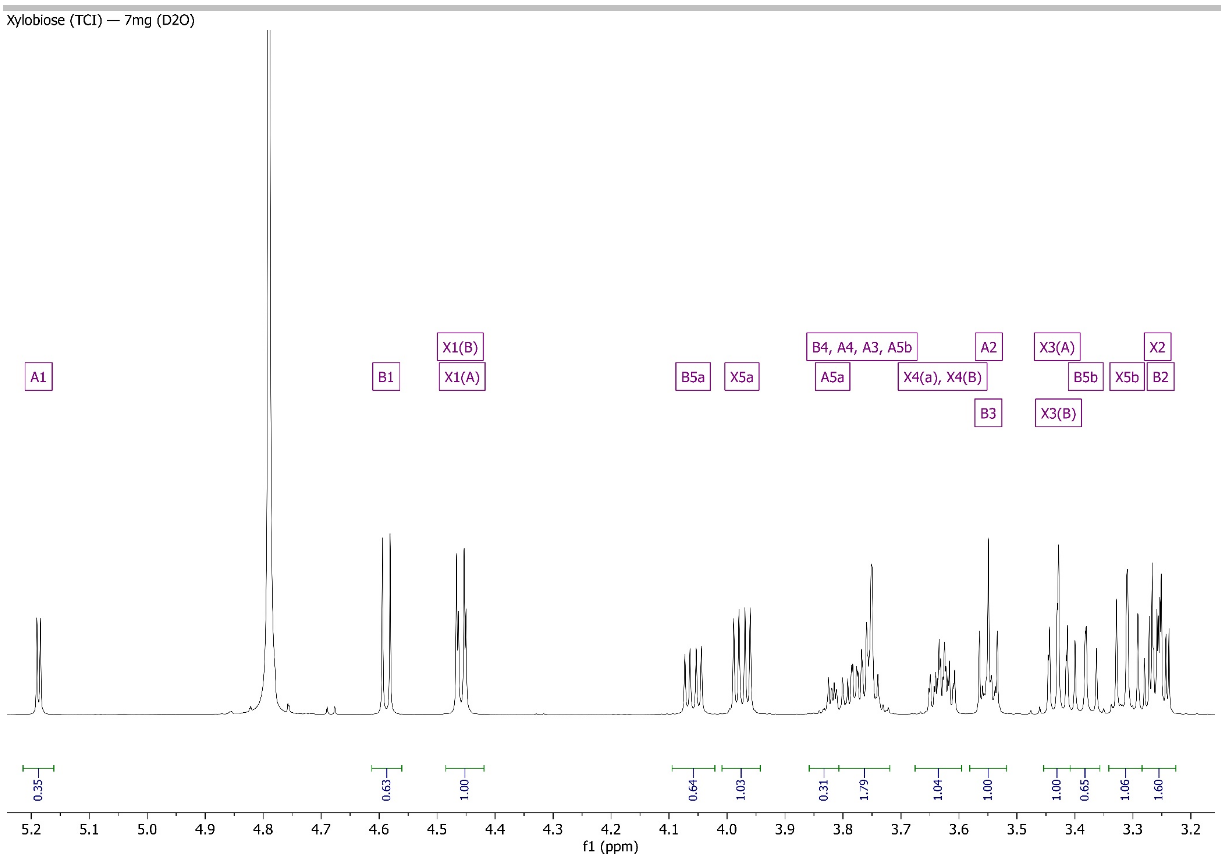

Figure S17. ^{1}H (600 MHz) spectra of commercial xylobiose in D_2O

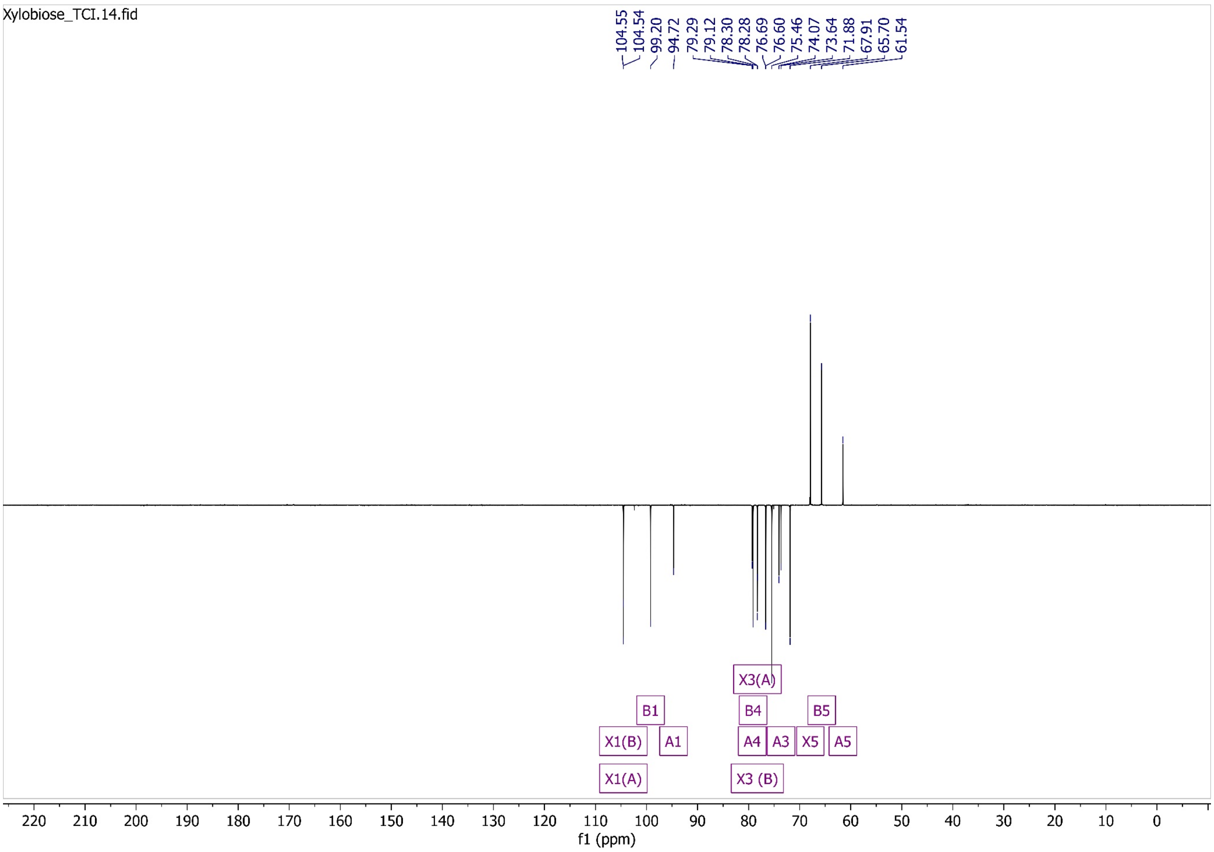

Figure S18. ^{13}C (151 MHz) spectra of commercial xylobiose in D_2O

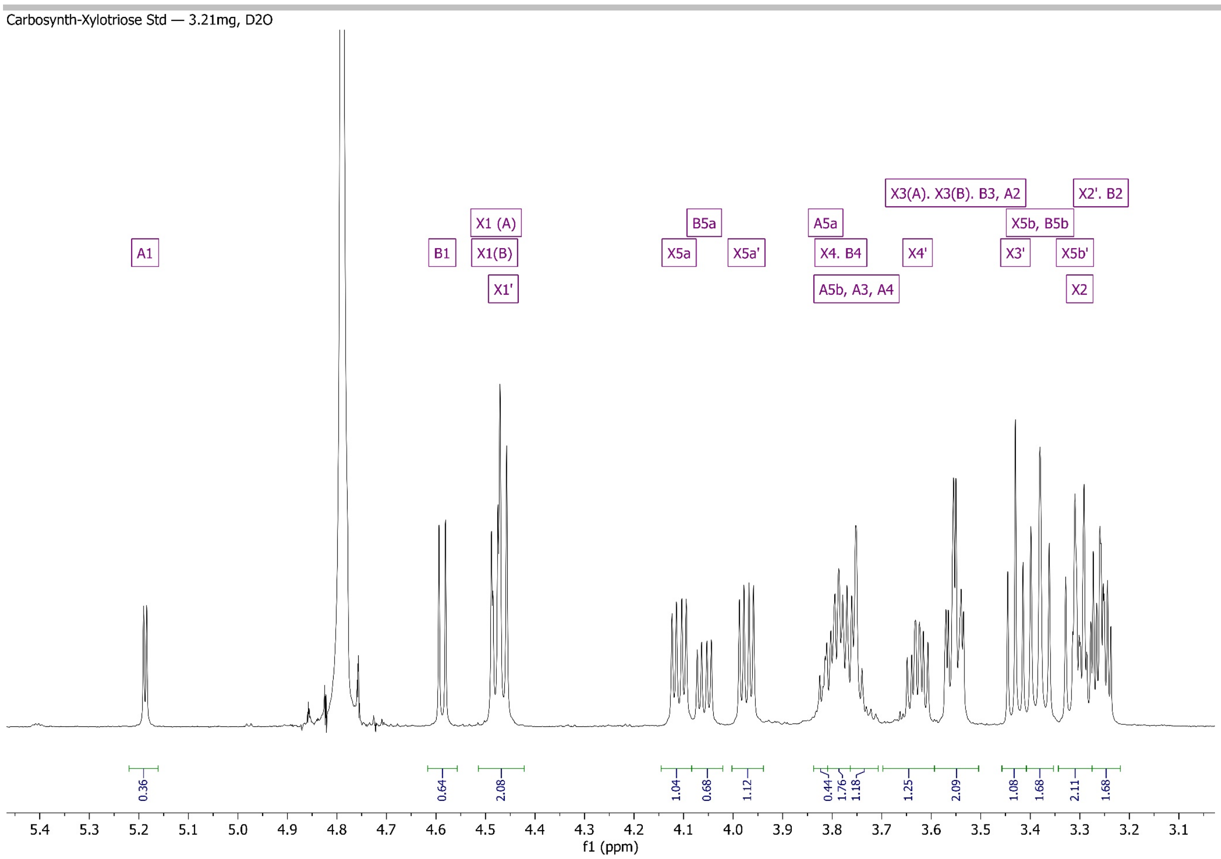

Figure S19. ^{1}H (600 MHz) spectra of commercial β-1,4-D-xylotriose in D_2O.

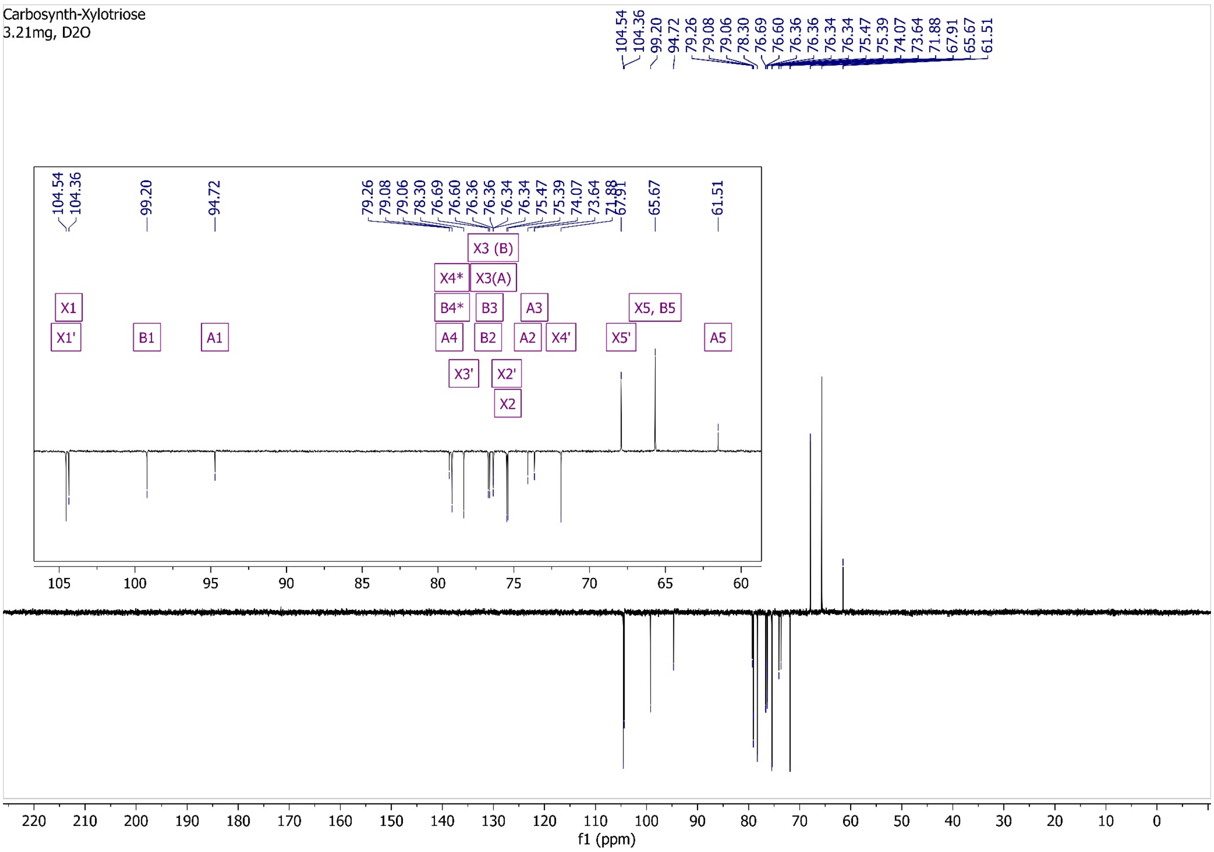

Figure S20. ^{13}C (151 MHz) spectra of commercial 1,4-β-D-xylotriose in D_2O.

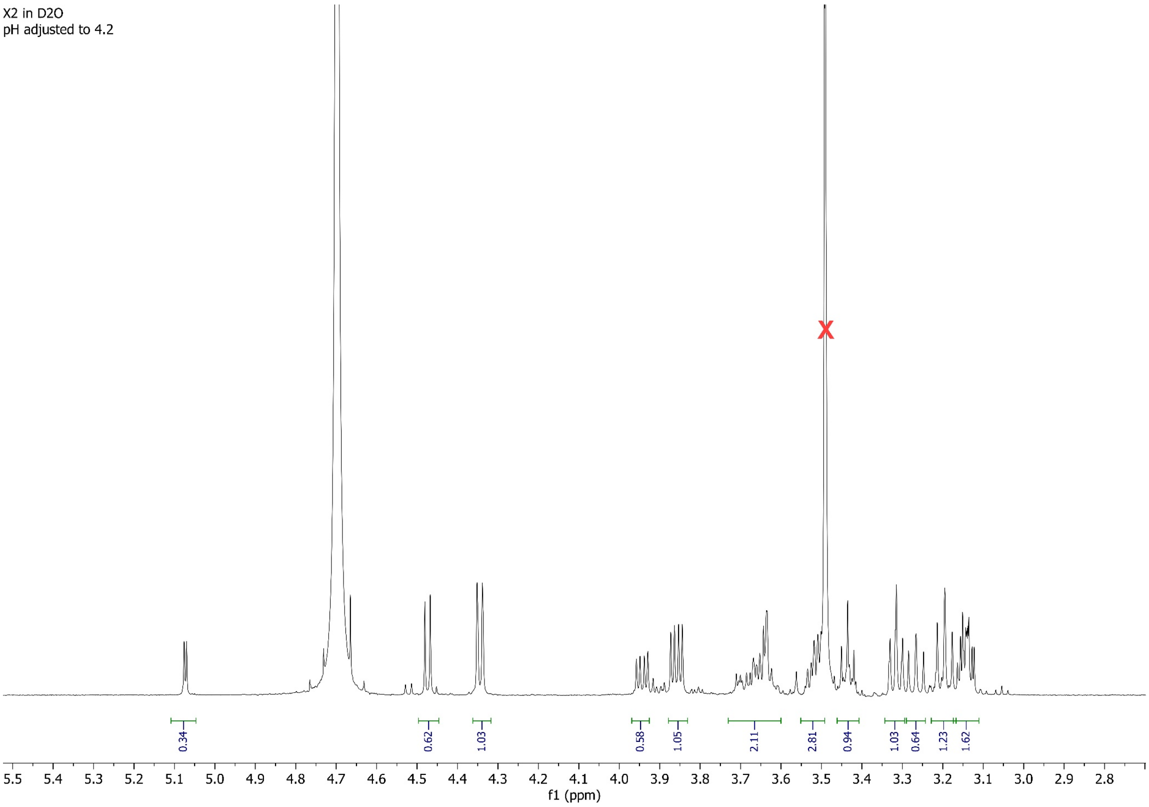

Figure S21. ^{1}H (600 MHz) spectra of SEC fraction X2

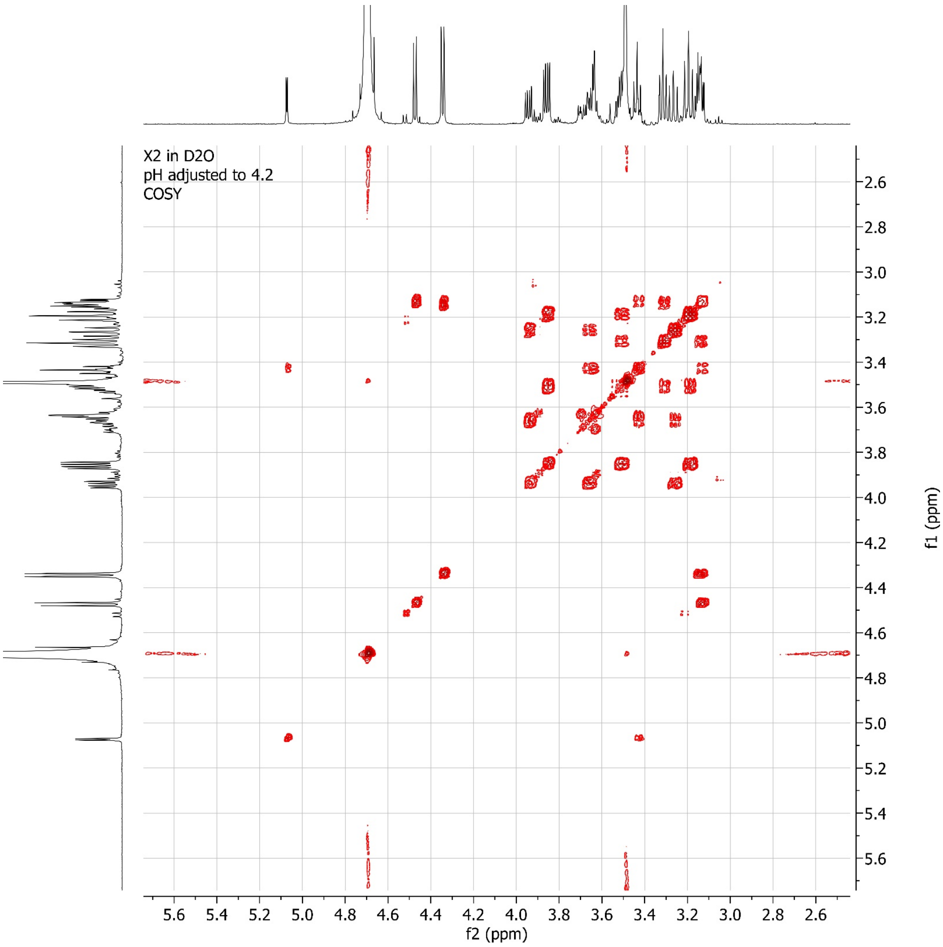

Figure S22. COSY spectra of SEC fraction X2

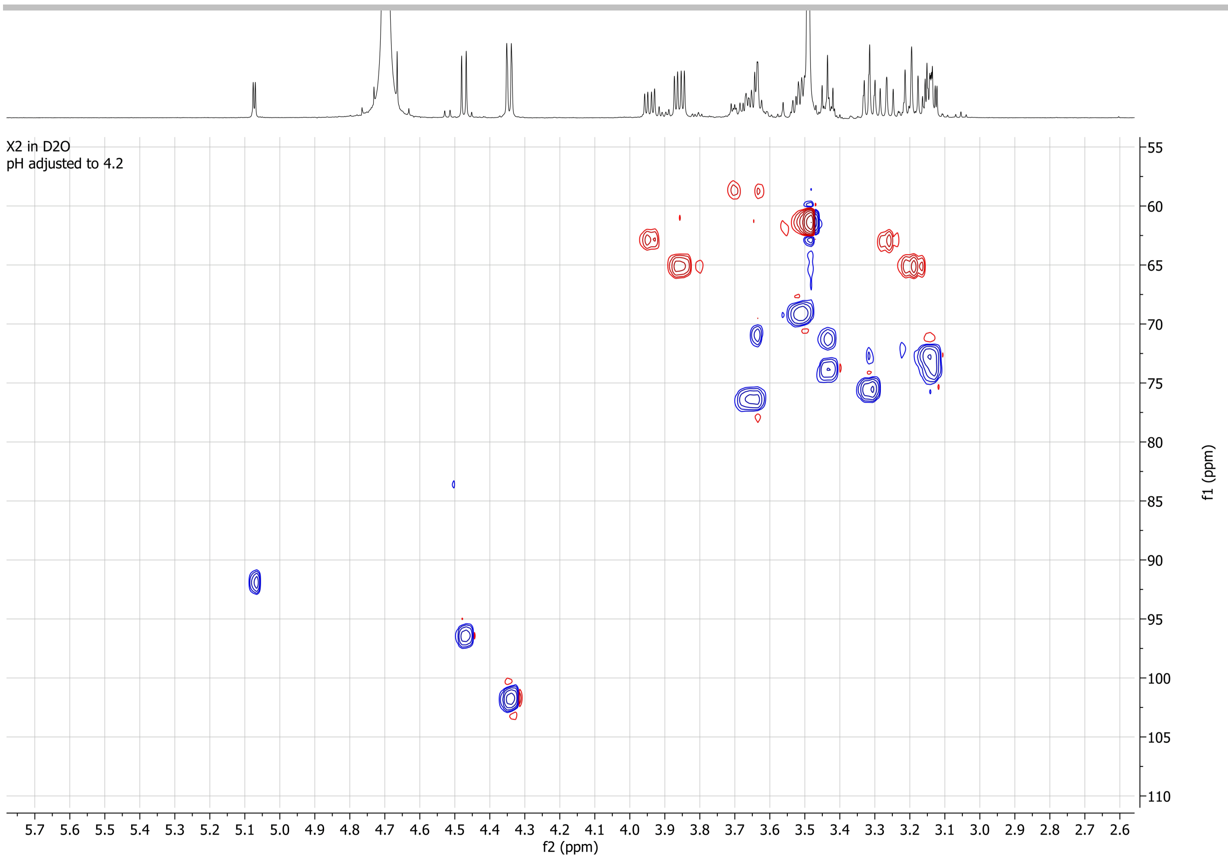

Figure S23. HSQC spectra of SEC fraction X2

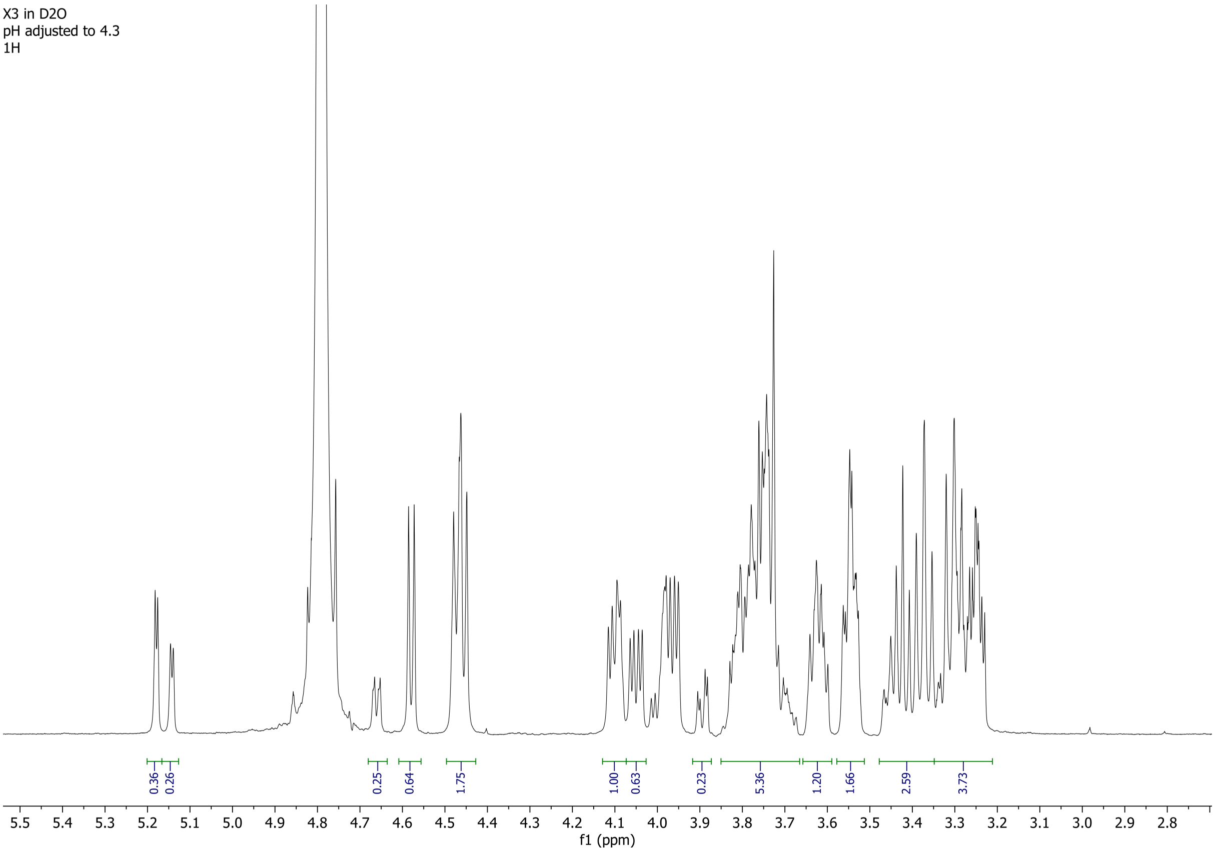

Figure S24. ^{1}H (600 MHz) spectra of SEC fraction X3

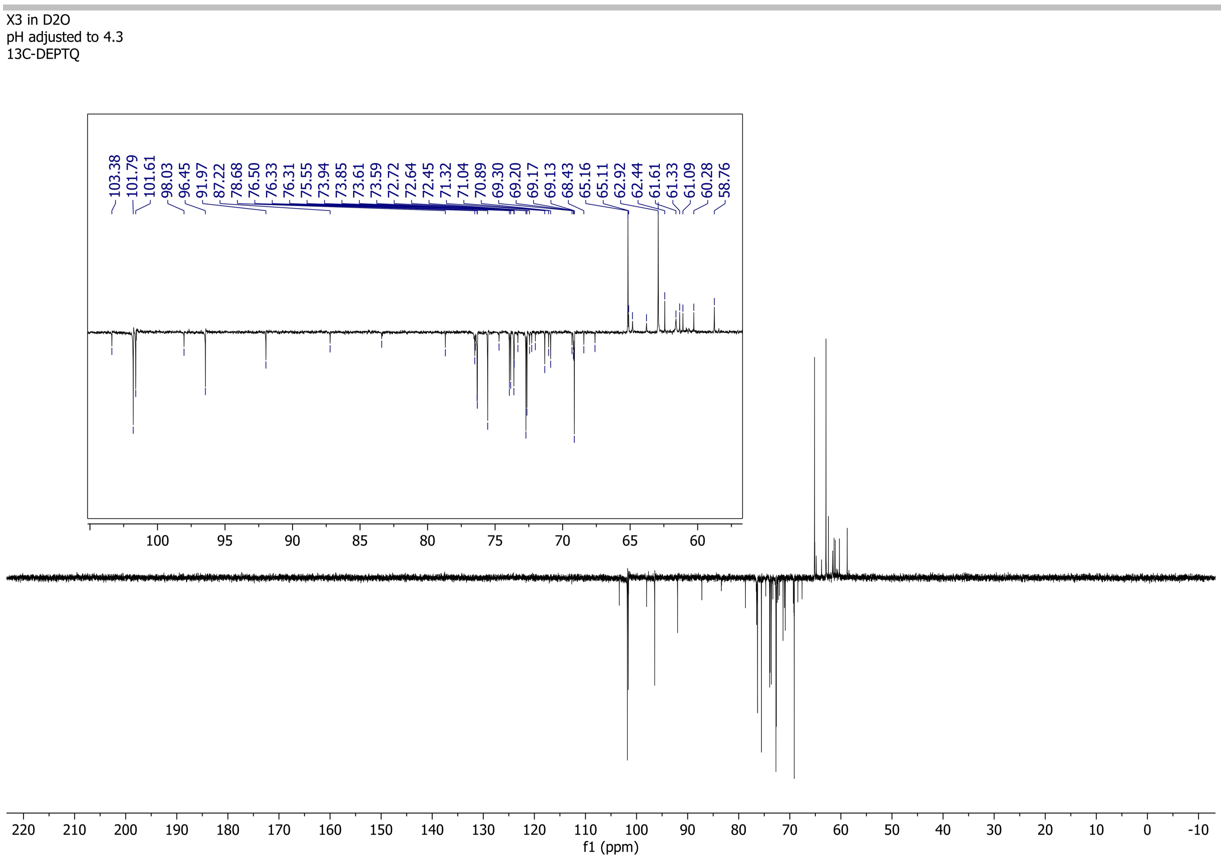

Figure S25. ^{13}C DEPTQ (141 MHz) spectra of SEC fraction X3

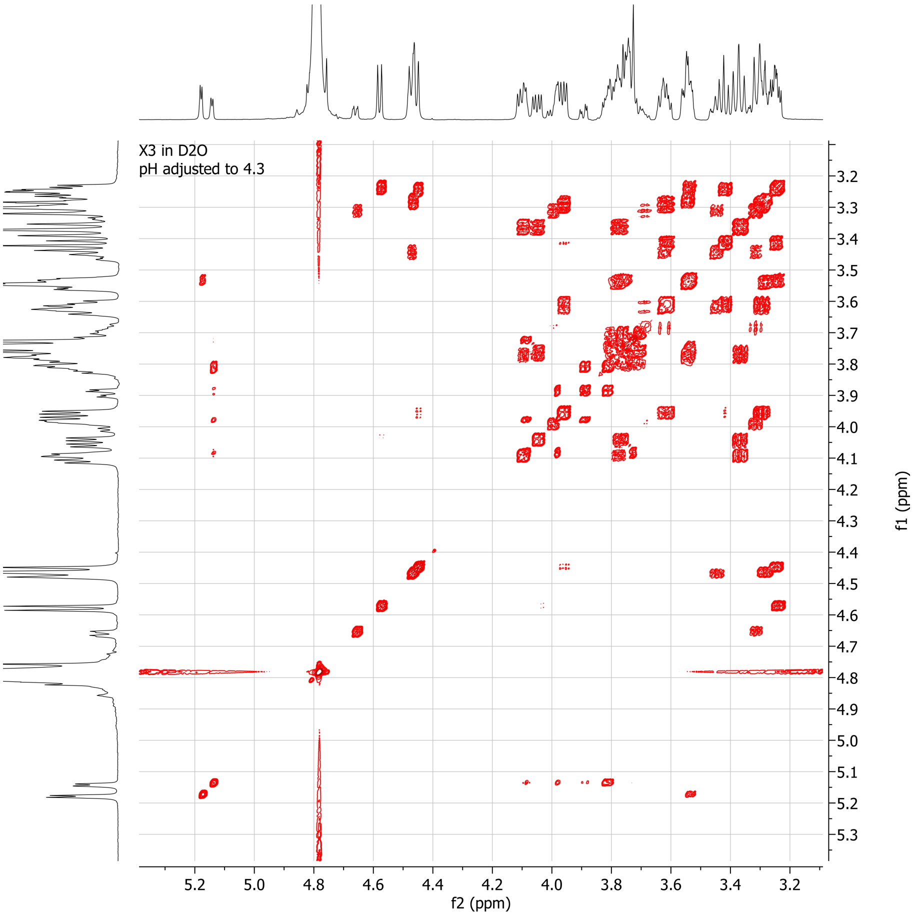

Figure S26. COSY spectra of SEC fraction X3

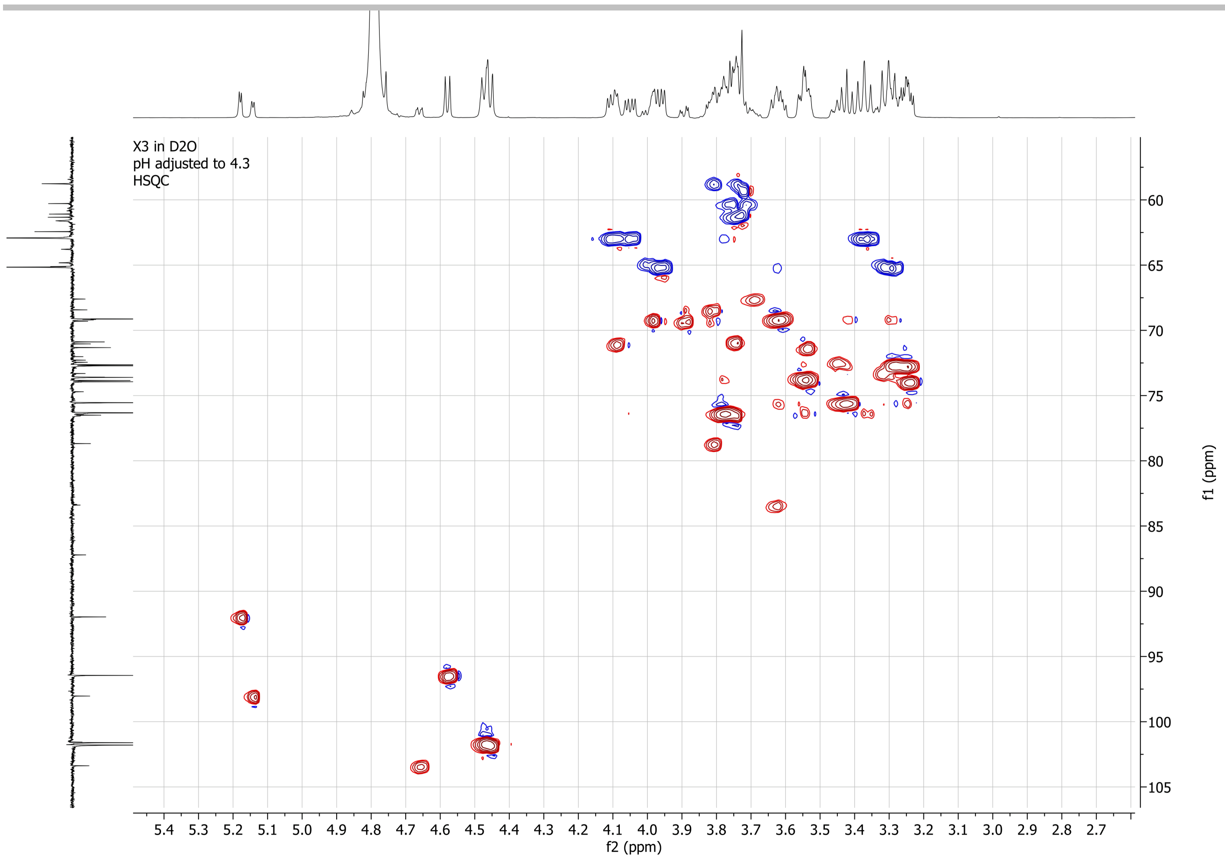

Figure S27. HSQC spectra of SEC fraction X3

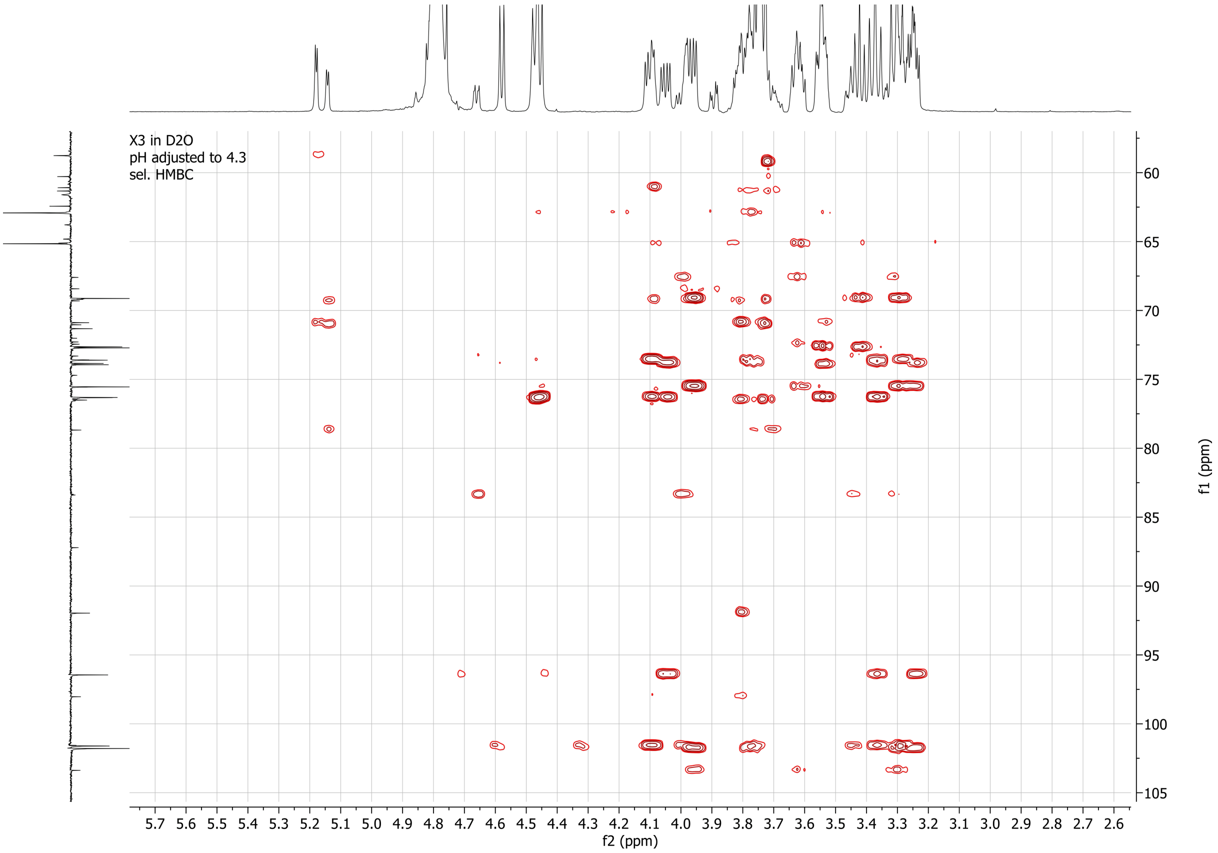

Figure S28. HMBC spectra of SEC fraction X3

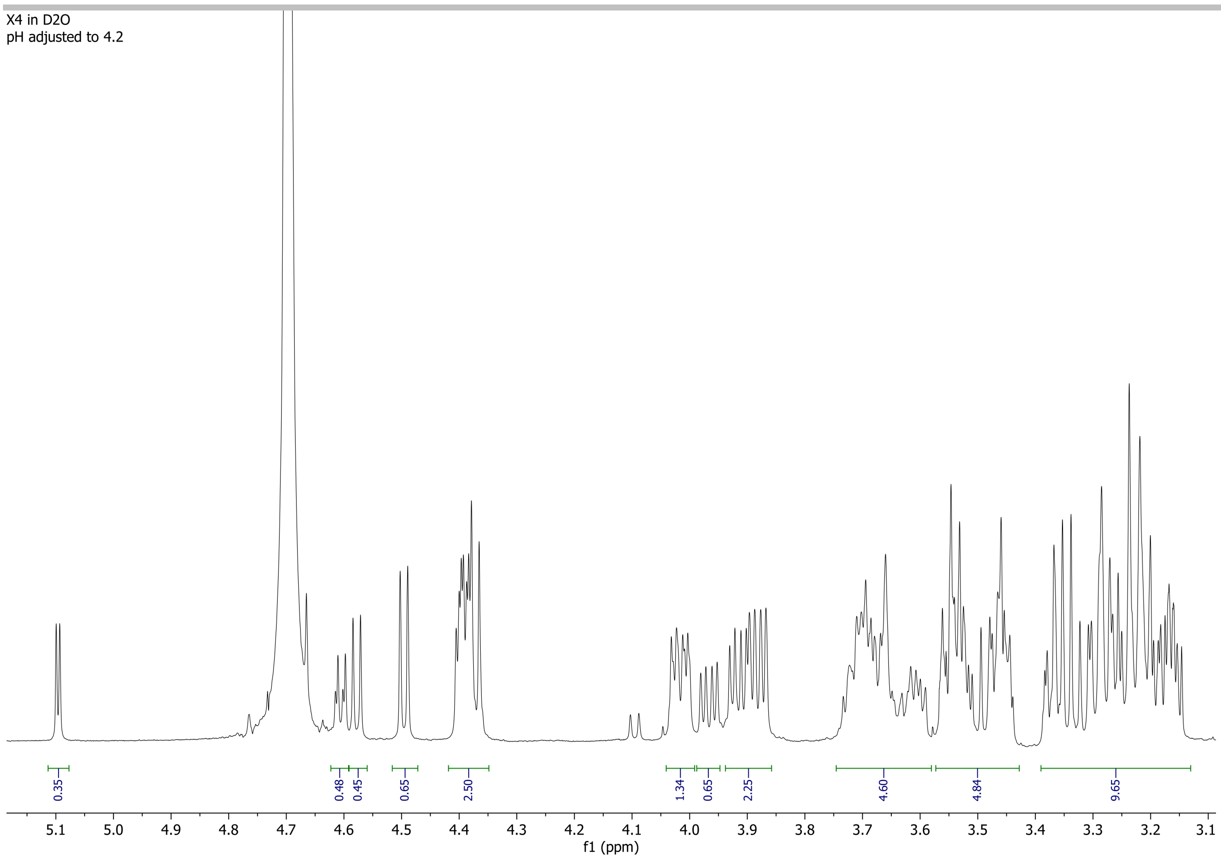

Figure S29. ^{1}H (600 MHz) of SEC Fraction X4 in D_2O

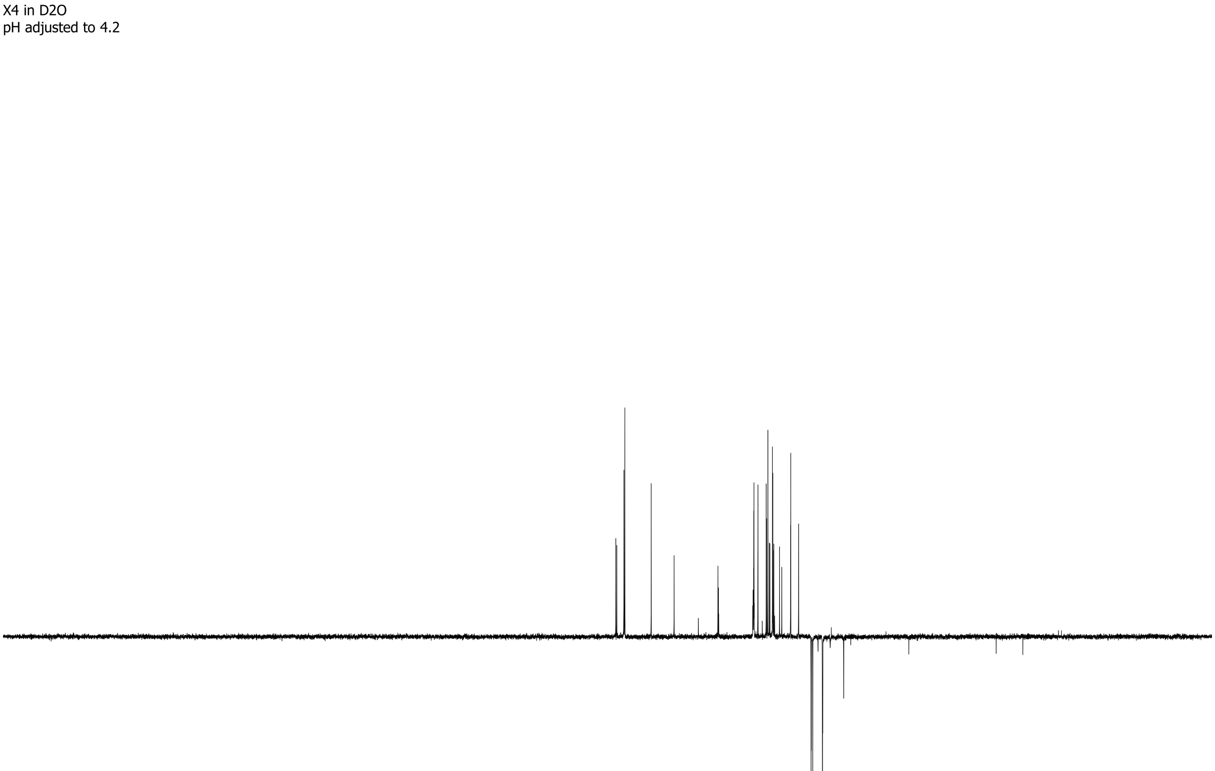

Figure S30. ^{13}C -DEPTQ (141 MHz) of SEC Fraction X4 in D_2O

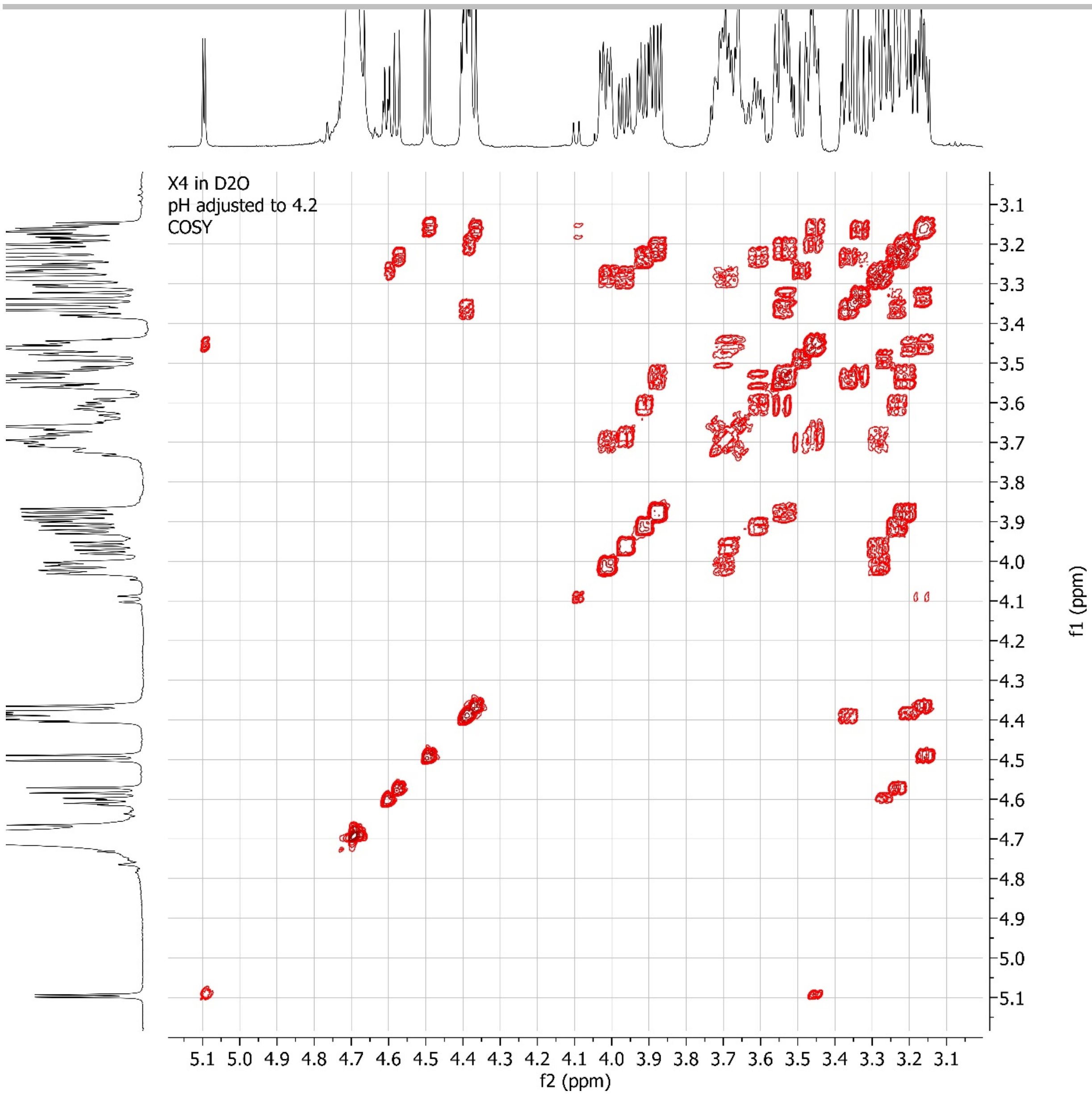

Figure S31. COSY spectrum of SEC Fraction X4 in D_2O

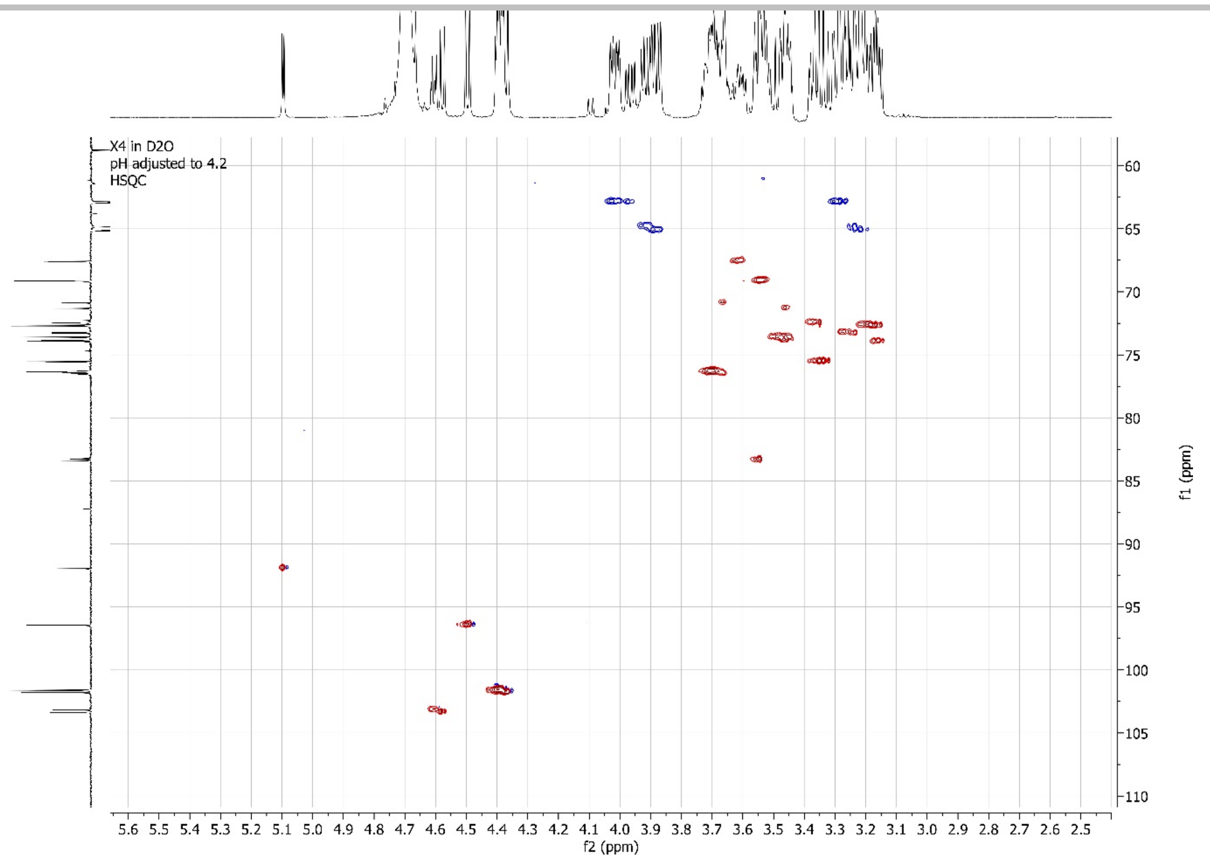

Figure S32. HSQC spectrum of SEC Fraction X4 in D_2O

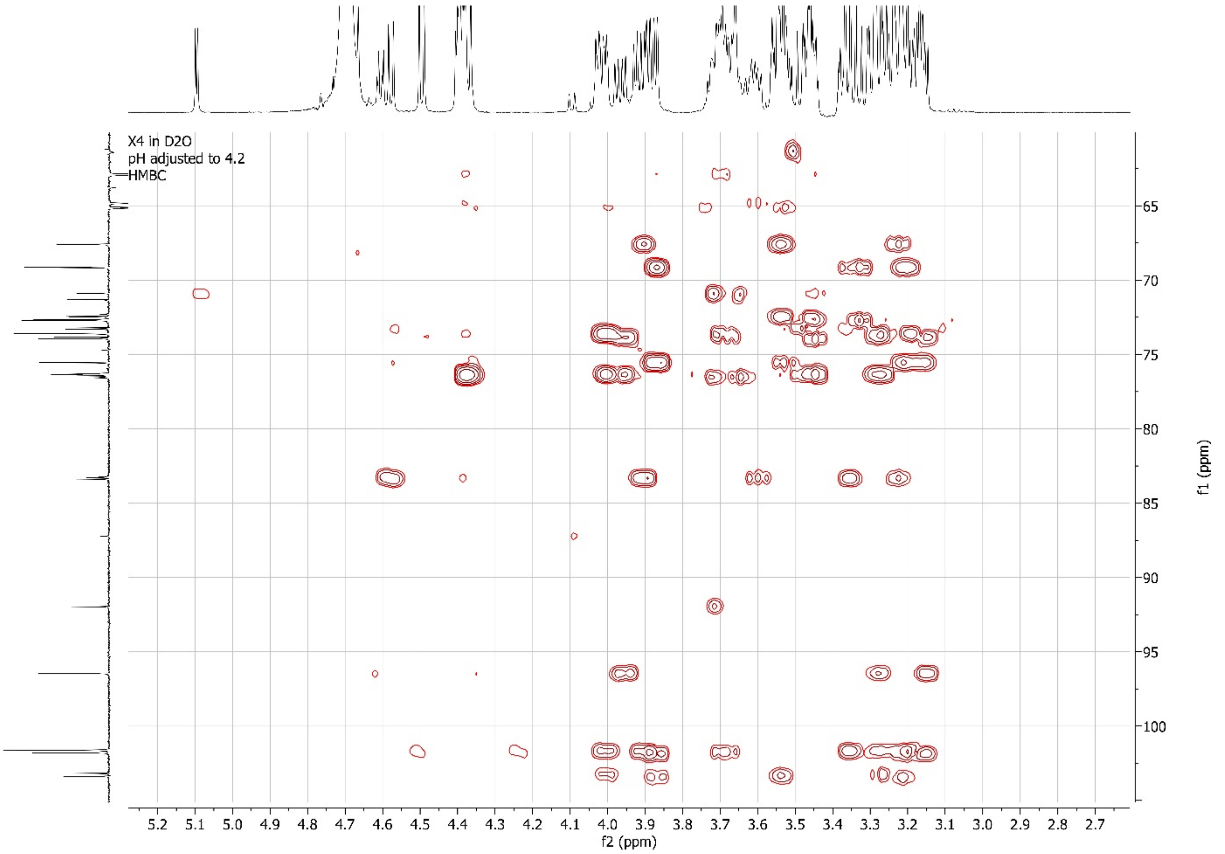

Figure S33. HMBC spectrum of SEC Fraction X4 in D_2O
